# Advanced backcross QTL analysis and comparative mapping with RIL QTL studies and GWAS provide an overview of QTL and marker haplotype diversity for resistance to Aphanomyces root rot in pea (*Pisum sativum*)

**DOI:** 10.3389/fpls.2023.1189289

**Published:** 2023-09-28

**Authors:** Théo Leprévost, Gilles Boutet, Angélique Lesné, Jean-Philippe Rivière, Pierrick Vetel, Isabelle Glory, Henri Miteul, Anaïs Le Rat, Philippe Dufour, Catherine Regnault-Kraut, Akiko Sugio, Clément Lavaud, Marie-Laure Pilet-Nayel

**Affiliations:** ^1^ IGEPP, INRAE, Institut Agro, University of Rennes, Le Rheu, France; ^2^ RAGT 2n, Druelle Balsac, France; ^3^ KWS MOMONT Recherche SARL, Allonnes, France

**Keywords:** *Aphanomyces euteiches*, sources of resistance, AB populations, integrative study, SNPs

## Abstract

*Aphanomyces euteiches* is the most damaging soilborne pea pathogen in France. Breeding of pea resistant varieties combining a diversity of quantitative trait loci (QTL) is a promising strategy considering previous research achievements in dissecting polygenic resistance to *A. euteiches*. The objective of this study was to provide an overview of the diversity of QTL and marker haplotypes for resistance to *A. euteiches*, by integrating a novel QTL mapping study in advanced backcross (AB) populations with previous QTL analyses and genome-wide association study (GWAS) using common markers. QTL analysis was performed in two AB populations derived from the cross between the susceptible spring pea variety “Eden” and the two new sources of partial resistance “E11” and “LISA”. The two AB populations were genotyped using 993 and 478 single nucleotide polymorphism (SNP) markers, respectively, and phenotyped for resistance to *A. euteiches* in controlled conditions and in infested fields at two locations. GWAS and QTL mapping previously reported in the pea-Aphanomyces collection and from four recombinant inbred line (RIL) populations, respectively, were updated using a total of 1,850 additional markers, including the markers used in the Eden x E11 and Eden x LISA populations analysis. A total of 29 resistance-associated SNPs and 171 resistance QTL were identified by GWAS and RIL or AB QTL analyses, respectively, which highlighted 10 consistent genetic regions confirming the previously reported QTL. No new consistent resistance QTL was detected from both Eden x E11 and Eden x LISA AB populations. However, a high diversity of resistance haplotypes was identified at 11 linkage disequilibrium (LD) blocks underlying consistent genetic regions, especially in 14 new sources of resistance from the pea-Aphanomyces collection. An accumulation of favorable haplotypes at these 11 blocks was confirmed in the most resistant pea lines of the collection. This study provides new SNP markers and rare haplotypes associated with the diversity of Aphanomyces root rot resistance QTL investigated, which will be useful for QTL pyramiding strategies to increase resistance levels in future pea varieties.

## Introduction

Genetic resistance represents a key approach to reduce chemical applications for sustainable crop disease management. Partial resistance, also known as “quantitative resistance”, is often governed by multiple QTL and characterized by a compatible interaction between the pathogen and its host plant, typically resulting in a reduction of disease severity and limited progression of the pathogen within the host tissues (Poland et al., 2009). Quantitative resistance has generally lower resistance effect but is considered more durable than monogenic complete resistance. Pyramiding a diversity of resistance QTL showing a broad spectrum of action on pathogen populations and targeting various steps in the pathogen life cycle appears to be a promising approach to increase the level and durability of quantitative resistance in plant breeding programs (Pilet-Nayel et al., 2017). Linkage analysis has been broadly employed to identify resistance QTL in RIL plant populations derived from single biparental crosses between parents showing contrasted level of resistance (Varshney and Dubey, 2009). Balanced allele segregation ratios and high recombination events enable efficient QTL detection in RIL populations but considerably delay the transfer of valuable resistance alleles from wild donor genotypes to elite breeding lines by backcrossing and/or intercrossing (Tanksley and Nelson, 1996). AB populations derived by backcrossing the F_1_ hybrid to the elite parent until an advanced generation, *e.g.* BC_2_ or BC_3_, allow to develop recombinant lines genetically less similar to the donor parental line, accelerating the transfer of wild alleles into agronomic lines. Transfer of disease resistance traits has been successfully achieved through AB-QTL analysis in several major field crops, like barley (Haas et al., 2016), maize (Palanichamy and Smith, 2022), rice (Jiang et al., 2020), sunflower (Talukder et al., 2022), and wheat (Naz et al., 2015). GWAS is a powerful tool to investigate complex genetic determinism and identify exotic or agronomic alleles in plant natural diversity panels. Compared to linkage analysis, GWAS takes advantage of high recombination rates between unrelated individuals to better refine genomic regions associated with trait variation. But, GWAS suffers also from low statistical power to detect low-frequency favorable alleles, *i.e.* carried by only a few genotypes of interest in the plant panel (Tibbs Cortes et al., 2021). Combining linkage analysis and GWAS is a promising approach to better understand polygenic determinisms underlying partial resistance to pathogens in plants.

Pea (*Pisum sativum*) is an important crop with significant nutritional and environmental value. It offers high protein (≈23.5%), vitamin, mineral, and carbohydrate-rich seeds for human and animal consumption. It also contributes to reduce nitrogen fertilization, due to its ability to fix atmospheric nitrogen through its symbiosis with soil bacteria, and to break disease cycles in cereal rotations (Amarakoon et al., 2012; Powers and Thavarajah, 2019). Aphanomyces root rot, caused by the soil-borne oomycete *Aphanomyces euteiches* Drechs., is among the most damaging pulse root rot diseases worldwide, particularly affecting spring pea varieties (Bénézit et al., 2017; Jha et al., 2021). The pathogen has been reported in 21 different countries across all five continents, including major pea-growing nations such as Russia, Canada, China, India, France, Australia, and the USA (Becking et al., 2022). Two main pathotypes of *A. euteiches* were reported, including pathotype I predominant in Europe and main pea-growing regions in Canada, and pathotype III observed in some regions of the USA (Le May et al., 2018; Sivachandra Kumar et al., 2021). Under favorable weather conditions, both pathotypes can cause rotting of roots and epicotyls, resulting in yellow leaves and, in some cases, plant mortality. In addition, the disease can cause yield losses of up to 100% in highly infested fields (Hughes and Grau, 2007). Two main recommended prophylaxis methods are commonly advised: (i) assessing the level of soil infestation to avoid the pea crop in contaminated fields and (ii) implementing crop rotations with non-host or resistant crops to reduce the inoculum potential in the soil (Wu et al., 2019). While chemical and biological strategies have demonstrated limited efficacy in controlling the disease, and complete resistance to *A. euteiches* has not been reported in any pea cultivar, breeding for quantitative resistance is a promising approach to reduce pea yield losses caused by the root rot disease (Wu et al., 2019; Wu et al., 2021).

Since the early-2000s, genetic resistance to *A. euteiches* in pea has been well-explored. Using four pea RIL populations derived from the partially resistant parents PI180693, 552, 90-2131, and 90-2079, linkage mapping studies, mainly based on SSR markers, identified 27 meta-QTL associated with partial resistance to *A. euteiches* in controlled conditions and/or infested field nurseries in France and the USA. The meta-QTL covered seven main resistance QTL regions (Pilet-Nayel et al., 2002; Pilet-Nayel et al., 2005; Hamon et al., 2011; Hamon et al., 2013). In particular, two major-effect QTL *Ae-Ps4.5* and *Ae-Ps7.6* located on linkage groups (LGs) IV and VII, were associated with a high level of partial resistance to the strains Ae109 (pathotype III) and RB84 (pathotype I), respectively (Hamon et al., 2011; Hamon et al., 2013, Lavaud et al., submitted^1^). The five other main QTL regions, presenting lower effects, were named *Ae-Ps1.2* on LGI genetically close to the *Af* locus (leaf type), *Ae-Ps2.2* on LGII close to the *A* locus (anthocyanin production), *Ae-Ps3.1* on LGIII close to the *Hr* locus (photoperiod high-responsive flowering), *Ae-Ps4.1* on LGIV and *Ae-Ps5.1* on LGV close to the *R* locus (seed type). At *Ae-Ps2.2* and *Ae-Ps3.1*, resistance and late-flowering alleles derived from PI180693 were reported to be linked (Hamon et al., 2013). Resistance to *A. euteiches* in pea was thus suggested to be influenced by pleiotropy or genetic linkage involving plant morphology and phenology genes. In addition, GWAS achieved in a collection of 175 *Pisum sativum* lines, mentioned as the “pea-Aphanomyces collection”, detected 52 resistance LD blocks associated with partial resistance to *A. euteiches*, which validated six of the seven main QTL previously reported (Desgroux et al., 2016). A QTL analysis conducted in Canada, from a pea RIL population whose resistant parent (00-2067) shares the same PH14-119 progenitor as 90-2079 (Kraft et al., 1972; Kraft, 1981; Kraft, 1992; Conner et al., 2013), also revealed a major-effect QTL which was identified in both field and greenhouse experiments. This QTL was found to be located near the *Ae-Ps4.5* region (Wu et al., 2021). Finally, using near-isogenic lines (NILs) carrying resistance alleles at different combinations of one to three of the seven main resistance QTL, major-effect and some minor-effect QTL were validated in controlled conditions (Lavaud et al., 2015; Lavaud et al., 2016) and infested French field nurseries (Lavaud et al., unpublished data). However, although the main Aphanomyces resistance QTL are currently used in research and pea private breeding programs, levels of partial resistance are still difficult to increase and the durability of major-effect QTL remains questioned (Quillévéré-Hamard et al., 2021). Thus, the identification of new QTL or alleles conferring resistance to *A. euteiches* would be useful, in order to diversify and cumulate resistance alleles for breeding pea varieties for high level and durable resistance.

From 2002 to 2008, a large germplasm screening program of approximately 1900 *Pisum* accessions was conducted in controlled conditions for resistance to *A. euteiches* and resulted in the selection of 20 partially resistant pea lines as new sources of resistance (Pilet-Nayel et al., 2007; Desgroux et al., 2016). Among the 20 pea lines, two exotic sources of resistance, named E11 and LISA, showed rare resistance haplotypes mostly different from the ones previously reported in the four RIL resistant parents (Desgroux et al., 2016). Therefore, E11 and LISA were crossed with the susceptible pea variety Eden to produce two pea AB populations, in order to simultaneously detect and introgress into an agronomic background new potential resistance QTL or alleles.

The objectives of this study were to (i) identify genetic loci controlling Aphanomyces root rot resistance in two new pea sources of resistance, then (ii) conduct a comparative genetic mapping of QTL identified in this study along with QTL updated from previous reports using common SNP markers. This comprehensive approach aimed to represent the diversity of QTL and haplotypes associated with resistance to *A. euteiches* on a reference consensus marker map. QTL mapping was carried out in the two new AB populations produced from the crosses Eden x E11 and Eden x LISA, using SNP marker genotyping data and Aphanomyces root rot resistance data collected in controlled conditions and infested field nurseries at two locations. Then, genetic analyses of resistance to *A. euteiches* previously conducted by linkage analysis and GWAS in the four RIL populations Baccara x PI180693, Baccara x 552, DSP x 90-2131, and Puget x 90-2079 and in the pea-Aphanomyces collection, respectively, were updated using new genotyping data from SNP markers common to both Eden x E11 and Eden x LISA AB populations. These common SNPs made it possible the comparison of QTL detected from the independent populations. By integrating GWAS, RIL, and AB QTL analyses in pea, this study identified consistent genetic regions and haplotypes of resistance to *A. euteiches*, for future pyramiding strategies of resistance alleles in pea breeding.

## Materials and methods

### Plant material

Two AB populations of 179 and 180 BC_2_F_7_ pea lines derived from the crosses Eden x E11 and Eden x LISA, respectively, were used for QTL mapping. Eden is a spring pea cultivar susceptible to *A. euteiches* with white flowers, afila leaves, and wrinkled seeds. E11 and LISA are two pea germplasms partially resistant to *A. euteiches*, showing high plants with purple flowers, normal leaves, and smooth seeds. LISA is a spring fodder cultivar which originates from Germany. The sowing type and end use of the wild Egyptian pea E11 remain unknown (Desgroux et al., 2016). Four pea RIL populations derived from the crosses Baccara x PI180693 (178 RILs), Baccara x 552 (178 RILs), DSP x 90-2131 (111 RILs), and Puget x 90-2079 (127 RILs), were used to update previous QTL mapping studies (Pilet-Nayel et al., 2002; Pilet-Nayel et al., 2005; Hamon et al., 2011; Hamon et al., 2013).

The pea-Aphanomyces collection of 175 lines previously described by Desgroux et al. (2016), was used for updated GWAS. The collection includes about 60% of spring-type germplasm lines, named AeA95xx, AeB97xx, AeD99xx, from an Aphanomyces recurrent breeding program conducted by French breeders in 1995-2005, using PI180693, 552 and 90-2131 as sources of resistance. It includes about 40% of best RILs, parental lines of mapping populations, sources of resistance selected from INRAE and USDA genetics programs for Aphanomyces root rot resistance, and French cultivars, described for different end-uses (food, feed or fodder peas) and sowing times (spring and winter peas). The pea lines in the collection showed different levels of resistance or susceptibility to *A. euteiches* and variability in agronomic traits (seed type, foliage type and flower color).

### Phenotyping

In infested fields, the Eden x E11 and Eden x LISA populations were evaluated for root rot disease resistance in two locations in France, *i.e.* Riec-sur-Belon, Finistère (RI) and Dijon-Epoisses, Côtes d’Or (DI) (Hamon et al., 2011), in 2019 and 2021, respectively. *A. euteiches* isolates of pathotype I were described from these infested fields (Quillévéré-Hamard et al., 2018; Onfroy et al, unpublished data). Field assays were carried out in the spring and sown in double-row plots of 30 plants/row. The plots were distributed according to a randomized complete block design with three replicates, incorporating crossed “assessor” et “gradient” blocks. A susceptible cultivar (Solara) was repeated every four plots to adjust disease severity score of the pea lines relative to that of the adjacent Solara plots, as described by Hamon et al. (2011). Two disease criteria were used to assess root rot resistance for each plot: (i) the root rot index (RRI), as the average disease severity score of 10 plants on a 0 (healthy plant) to 5 (dead plant) scoring scale, evaluated in Riec-sur-Belon, and (ii) the aerial decline index (ADI), as the disease impact score on all the plants in the plot, on a 1 (green plant) to 8 (dead plant) scoring scale, evaluated in Riec-sur-Belon and Dijon-Epoisses, as described by Hamon et al. (2011). The number of calendar days to 50% bloom (Flo1) was also evaluated in Dijon-Epoisses for each plot.

In inoculated controlled conditions, the Eden x E11 and Eden x LISA populations were evaluated for root rot resistance to both pure-culture strains (i) *A. euteiches* Ae109 (pathotype III) and (ii) *A. euteiches* RB84 (pathotype I), respectively. For each strain and population, all the AB lines and parents were evaluated within a single disease test comprising four randomized complete blocks with five plants/block, as described by Moussart et al. (2001). Disease severity was scored on each plant using the same 0 (healthy plant) to 5 (dead plant) scoring scale as used for the field scorings (Hamon et al., 2011).

Phenotyping datasets produced on the RIL populations by Pilet-Nayel et al. (2002; 2005); Hamon et al. (2011; 2013) and Lavaud et al. (submitted^1^), and datasets produced on the pea-Aphanomyces collection by Desgroux et al. (2016), were used in this study.

### Statistical analysis of phenotypic data

Phenotypic datasets for plant morphology and resistance to *A. euteiches*, obtained from the Eden x E11 and Eden x LISA populations, were analyzed using the R 4.0.2 software (R Core Team, 2020).

Global statistical analyses were conducted to assess: (i) genotype x environment interactions in field experiments, employing a global linear model [R function lm], and (ii) genotype x strain interactions in controlled conditions tests, utilizing a cumulative link model (R function clm of package ordinal; Christensen, 2019).

In addition, individual statistical analyses of phenotypic data obtained from each field and controlled conditions experiment were computed using (i) a linear mixed model (R function lmer of package lme4; Bates et al., 2019) for field variables, including G (genotype) as fixed factor and replicates with assessor (R_A_) and gradient (R_Gr_) blocks, as random factors, and (ii) a cumulative link mixed model (R function clmm of package ordinal; Christensen, 2019) for controlled conditions variables, including G as fixed factor, and B (block) and GxB interaction as random factors.

Broad-sense heritability (H^2^) was assessed for each variable from variance estimates in a global linear model, including only G as fixed factor, and using the formula: H² = σ_G_^2^/[σ_G_^2^ + (σ_E_^2^/r)], where σ_G_^2^ is the genetic variance, σ_E_^2^ the residual variance and *r* the number of replicates per genotype.

Significance of factor effects in each model was tested (R function Anova of package car and RVAideMemoire; Fox and Weisberg, 2020; Hervé, 2020) and estimated marginal means (EMMs) were computed for all individual variables on each genotype and from each model (R function and package emmeans; Lenth, 2020). Pearson correlation analysis was carried out between EMMs (R function rcorr of package Hmisc; Harrell and Dupont, 2020) and a correlation matrix was drawn using the Pearson coefficient (r; R function rcorr of package corrplot; Wei and Simko, 2017).

Adjusted means datasets produced by Pilet-Nayel et al. (2002; 2005); Hamon et al. (2011; 2013) and Desgroux et al. (2016) were used for updated QTL mapping and GWAS, respectively.

### Genotyping

The Eden x E11 AB population was genotyped using a total of 1,850 markers designed in KASP™ assays as described in Boutet et al. (2016). The 1,850 markers were selected from Duarte et al. (2014); Boutet et al. (2016) and Tayeh et al. (2015), based on their genetic positions regularly distributed outside and inside stress resistance QTL. In the Eden x E11 population, 1,010 markers were retained as polymorphic, including 725, 246, and 37 SNP markers named “Ps1” (Boutet et al., 2016), “PsCam” (Tayeh et al., 2015) and “Ps0” or “Ps9” (Duarte et al., 2014), respectively, and two other SNPs located in trypsin inhibitor gene loci. The Eden x LISA AB population was genotyped using a sub-set of 481 polymorphic SNPs among the 1,850 markers, including 117 “Ps1”, 127 “PsCam” and 10 “Ps0” or “Ps9” common SNPs to the 1,010 markers selected in the Eden x E11 population. The Eden x E11 and Eden x LISA genotyping datasets were then reduced to 993 and 478 SNPs respectively, based on marker quality (heterozygosity ratio < 15%) and quality of linkage mapping (see next section) in each LG. A total of 160 and 178 Eden x E11 and Eden x LISA pea lines were selected, respectively, based on marker quality (missing data < 15% and heterozygosity ratio < 15%), quality of linkage mapping, and pea line homozygosity for flower color, foliar type, and plant morphology, observed in controlled conditions and greenhouse in Le Rheu, Ille-et-Vilaine, as well as in infested field nurseries in Riec-sur-Belon and Dijon-Epoisses.

Genotyping datasets produced on RIL populations by Pilet-Nayel et al. (2002; 2005) and Hamon et al. (2011; 2013), and genotyping dataset of 12,067 SNPs previously selected on the pea-Aphanomyces collection by Desgroux et al. (2016), were supplemented with the 1,850 markers which were used as bridges for comparative AB-, RIL- QTL mapping and GWAS. Genotyping matrices consisting of 1,866, 1,082, 950 and 669 SNPs and 176, 178, 111 and 121 RILs were established for Baccara x PI180693, Baccara x 552, DSP x 90-2131 and Puget x 90-2079 populations, respectively, based on quality of linkage mapping in each LG. A genotyping matrix consisting of 10,824 SNPs and 172 pea genotypes from the pea-Aphanomyces collection was retained, based on marker quality (markers with MAF > 5% and missing data < 10%; individuals with heterozygosity ratio < 15% and missing data < 10%). The filtered genotyping matrix, containing 0.94% missing values, was imputed using Beagle 5.1 software (Browning et al., 2018). Imputation parameters were established using a sliding window, length of overlap between adjacent sliding windows, and number of iterations of 15 cM, 5 cM, and 15, respectively.

### Linkage mapping and consensus marker map construction

Genetic maps for AB and RIL populations were established using the “sem” and “annealing 100 100 0.1 0.9” commands of CarthaGene software (de Givry et al., 2005), as presented in Boutet et al. (2016). These commands allowed the computation and optimization of the maximum likelihood for the order and position of markers (in cM Haldane) on each LG. For each SNP marker from each AB or RIL genetic map, a χ^2^ test (*p-value* < 0.001) was used to analyze adjustments of allelic segregation to the expected Mendelian ratios (1:1 in RIL populations, 1:7 in BC_2_F_7_ populations).

A consensus marker map, named “DORA” as the project name of this study, including 16,647 markers, was obtained by projecting the positions of the 1,850 markers used as supplemental marker set in this study onto the pea reference consensus genetic map described by Tayeh et al. (2015), using Biomercator 4.2 software (Sosnowski et al., 2012). Pairwise LD (r^2^) between markers was explored within LGs using PLINK 1.9 software (Purcell et al., 2007; Chang et al., 2015), as described by Desgroux et al. (2016).

### AB and RIL populations QTL analysis

Composite interval mapping models were performed using R/qtl package (Broman et al., 2003) to identify QTL for resistance to *A. euteiches*, flowering, and morphological traits from the RIL and AB populations. For each variable, a forward-backward stepwise selection of QTL covariate (window size = 5cM) was performed using the Haley-Knott regression method. To limit the number of covariables and potential impacts of overparameterization, new LOD score thresholds were estimated for each additional covariable introduced in the composite interval mapping models. These thresholds were computed by conducting 1,000 permutations to determine, with a genome-wide α error risk of 5%, the significance of putative QTL. For each QTL, the percentage of phenotypic (R^2^) and genotypic variation, additive and epistatic interaction effects, and one LOD drop-off confidence intervals were computed.

### Population structure, individual relatedness, and genome-wide association study

The structure of the collection was investigated using the ADMIXTURE 1.3 software (Alexander et al., 2009) with the whole SNP dataset. Ten groups were determined after 15 cross-validations according to the procedure presented in ADMIXTURE. The structure matrix was represented using pophelper (Francis, 2017). IBD relatedness between individuals was computed using the Astle and Balding (2009) algorithm. Ward’s clustering of pea lines was computed according to their estimating IBD relatedness values and was represented with the kinship matrix using GAPIT R packages (Lipka et al., 2012).

GWAS was performed using a modified version of the multi-locus mixed model (MLMM) R package (Segura et al., 2012), as described by Desgroux et al. (2016). For each variable, a forward-backward regression model (maxsteps = 5) was performed to select significant SNP markers as covariates. To declare significant SNPs, a multiple-Bonferroni (mBonf) threshold of 4.44 (*p-value* of 3.6E-05) was calculated using the formula: mBonf = [−log(α/m)] described by Desgroux et al. (2016), with α = 10%, the overall false positive threshold, and m = 2,738, the number of markers selected at non-redundant genetic positions on the DORA consensus marker map. The structure and kinship matrices were also set as covariates in MLMM models. For each model, the *p-value* and allelic effect of significant SNPs, and the partition of variance explained by covariates were scored.

### Comparative mapping

Genetic maps, QTL identified in this study from linkage analysis of AB and RIL populations, and QTL recently detected for resistance to the Ae109 and RB84 strains in the Puget x 90-2079 RIL population (Lavaud et al., submitted^1^) were projected onto the consensus marker map DORA using Biomercator 4.2 software. QTL and marker-trait associations identified by linkage analysis and GWAS, respectively, were visualized on the consensus marker map DORA using MapChart 2.1 software (Voorrips, 2002). Consistent genetic regions controlling partial resistance to *A. euteiches* were defined according to the colocalization of at least four partial resistance QTL and/or resistance-associated SNPs identified by linkage analysis and/or GWAS, respectively, based on their genetic positions on the consensus genetic map. Intervals of consistent genetic regions were delimited by the positions of upper and lower resistance QTL or resistance-associated GWAS-SNPs overlapping at least one of the four co-located resistance QTL/SNPs of each region. Main *Ae-Ps* QTL identified by Hamon et al. (2011; 2013) were repositioned using genetic analysis results and DORA marker map positions.

### Haplotype analysis

Local LD analysis was performed to define LD block intervals around significant markers detected by GWAS in consistent genetic regions of partial resistance, using PLINK 1.9 software. A LD block was determined as the interval including all markers in LD (r^2^ > 0.7) with the targeted marker. For each variable associated with a significant marker in a given LD block, EMMs of pea line groups carrying haplotypes that were not considered as rare, *i.e.* comprising more than 8 individuals (> 5% of the total number of accessions), were computed and compared using the Tukey-HSD test (α = 5%; R functions emmeans and cld of package emmeans). For each LD block, haplotypes significantly associated with higher or lower mean phenotypic scores compared to the other haplotypes, were defined as favorable (named a) or unfavorable (named b or c), respectively. At each LD block containing most significant markers, the phenotypic mean and range (adjusted EMMs) of the group of pea lines carrying different rare haplotypes (cumulated frequency > 5%; n ≥ 2 rare haplotypes) was compared to that of the group of pea lines carrying the favorable or unfavorable haplotype, using the Tukey-HSD test (α = 5%). Results of mean comparison tests were shown using box plots (R function ggplot of package ggplot2; Wickham, 2016).

## Results

### Phenotypic data analysis

In both Eden x E11 and Eden x LISA AB populations, global statistical analyses of disease scores obtained in field and controlled conditions experiments revealed significant genotype x environment (*p-value* < 0.05) and highly significant genotype x strain (*p-value* < 0.001) interaction effects, respectively, except for the first ADI ratings evaluated in Riec-sur-Belon and Dijon-Epoisses in the Eden x E11 population. The analysis of phenotypic and QTL data was therefore carried out for each environment and strain.

Individual statistical analyses of all *A. euteiches* resistance and flowering traits evaluated in both AB populations displayed significant G effects (*p-value* < 0.01), except for the EL_RI21_RRI variable (*p-value* = 0.07). They showed significant R_A_ or R_Gr_ effect for field variables (*p-value* < 0.01), as well as significant B effects (*p-value* < 0.05) and GxB interactions (*p-value* < 0.001) for controlled conditions variables, except for the E11_Ae109 variable (*p-value* = 0.54 and 0.14, respectively). Broad-sense heritability of ADI variables ranged from very low values for the first ratings performed in both Eden x E11 and Eden x LISA populations in Riec-sur-Belon (H^2^ = 0.05 and 0.13, respectively) to high values for the third ratings in Dijon-Epoisses (H^2^ = 0.67 and 0.73, respectively). Heritability values for root resistance evaluated in Riec-sur-Belon and in controlled conditions ranged from 0.41 to 0.86 and were especially higher in the Eden x E11 population (H^2^ > 0.81) compared to those assessed in the Eden x LISA population (H^2^ < 0.61). Heritabilities of flowering traits were very high in each population (H^2^ > 0.95). Frequency distributions of EMMs values for each variable tended to fit normal curves except for flowering traits which showed bimodal distribution. Transgressive segregation was observed for all the traits (**Supplementary Figure 1**; **Supplementary Table 1**).

**Figure 1:**
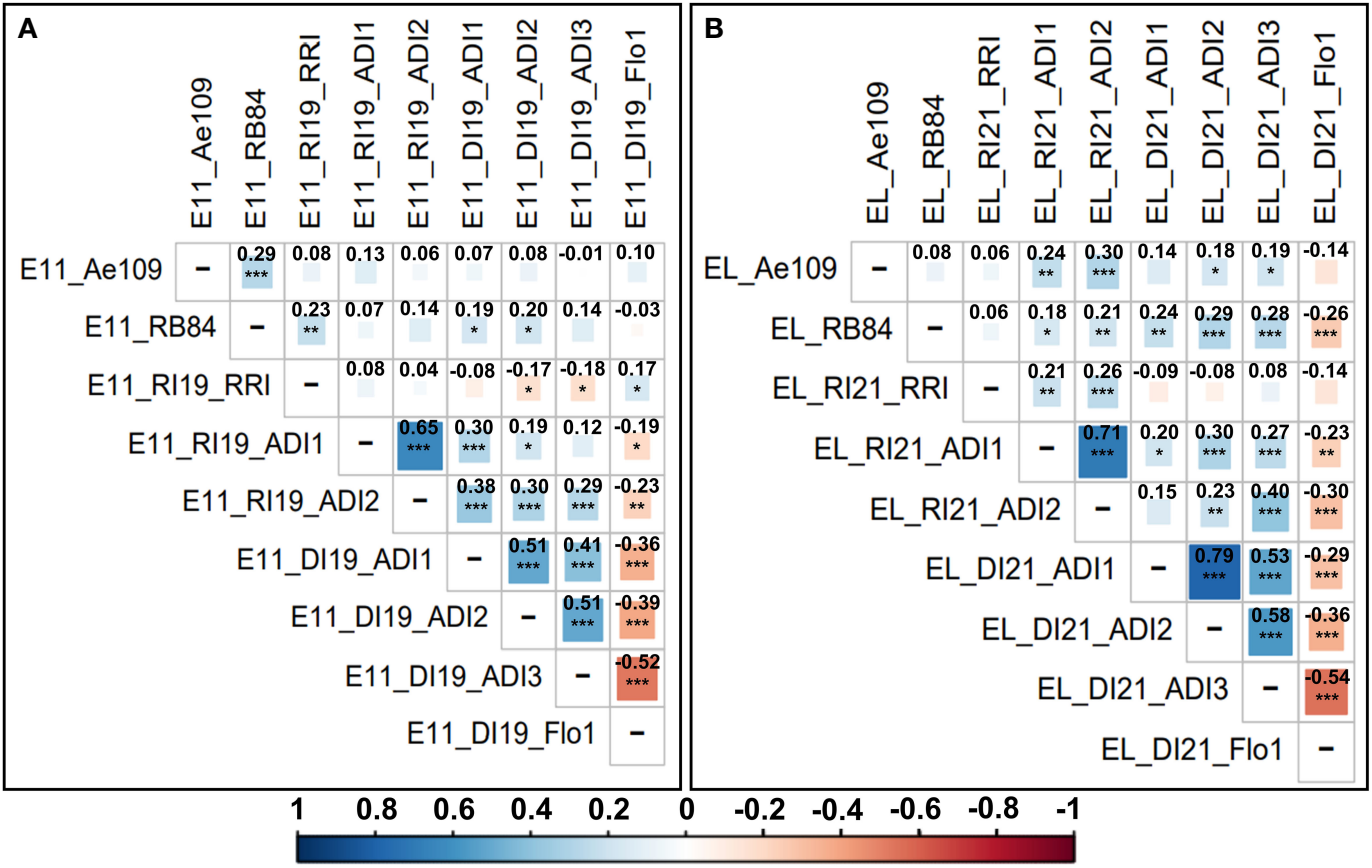
Correlogram and Pearson correlation coefficients between the different adjusted mean scoring data variables obtained for resistance to *A. euteiches* and flowering traits in the **(A)** Eden x E11 and **(B)** Eden x LISA AB populations. Scoring variables are coded as follows: population (E11 = Eden x E11 and EL = Eden x LISA); location (DI = Dijon-Epoisses, Côtes d’Or and RI = Riec-sur-Belon, Finistère (France)); experimental year (19 = 2019 and 21 = 2021); criterion (controlled conditions: Ae109 and RB84 = *A euteiches* strains belonging to the pathotype III and I, respectively; field: ADI = Aerial Disease Index, Flo1 = Number of calendar days to 50% bloom, RRI = Root Rot Index). Pearson correlation coefficients are indicated in bold. Level of correlation is coded with a color gradation scale from dark blue or red (r = ± 1) to white (r = 0). Significance *p-value* codes: 0 < ‘***’ ≤ 0.001 < ‘**’ ≤ 0.01 < ‘*’ ≤ 0.05.

In both AB populations, all ADI scores assessed in Riec-sur-Belon and Dijon-Epoisses were highly significantly and positively (r > 0.41, *p-value* < 0.001) correlated within each location, and most of the ADI variables showed significant and positive correlations between locations. RRI scores assessed in Riec-sur-Belon were poorly correlated with other field data except for ADI scores evaluated in the Eden x LISA population in the same environment (r > 0.21, *p-value* < 0.01). In the Eden x E11 population, RB84 and Ae109 strain data were significantly correlated between each other (r = 0.29, *p-value* < 0.001) but poorly correlated with all the other traits, except for the RB84 and RRI data (r = 0.23, *p-value* < 0.01). In the Eden x LISA population, RB84 and Ae109 data were not correlated between each other but were mostly significantly and positively correlated with ADI scores evaluated in Riec-sur-Belon and Dijon-Epoisses, especially for RB84 strain data (r > 0.18, *p-value* < 0.05). Flowering data were significantly (*p-value* < 0.05) and negatively (r < -0.19) correlated with ADI scoring data, and were poorly correlated with field RRI and controlled conditions data in both populations, except for the RB84 strain data (r < -0.26, *p-value* < 0.001) associated with the Eden x LISA population (**Figure 1**).

### Genetic maps construction

The genetic maps, constructed from the Eden x E11, Eden x LISA, Baccara x PI180693, Baccara x 552, DSP x 90-2131, and Puget x 90-2079 populations, comprised 993, 478, 1,866, 1,082, 950, and 669 markers covering 785.0, 449.6, 927.8, 982.3, 854.5, and 644.8 cM Haldane, respectively, over nine to nineteen LGs (**Table 1** and **Supplementary Table 2**). On each genetic map, marker distribution and order were well conserved with the pea reference consensus map presented in Tayeh et al. (2015). Depending on the RIL genetic map, the average marker densities ranged from 1.0 to 2.0 markers/cM and the maximum gaps between two contiguous markers varied from 13.0 to 18.1 cM. Although the AB populations exhibited lower levels of genetic recombination compared to the RIL populations, the quality of linkage mapping remained satisfactory. Specifically, the Eden x E11 and Eden x LISA genetic maps displayed an average marker density of 1.3 and 1.1 markers/cM, respectively, along with a maximum gap of 18.2 and 10.9 cM, respectively. Non-Mendelian allelic segregation (α = 0.001) was observed for less than 5.5% of the total number of markers in each RIL and AB population, except for the Eden x E11 AB population showing 214 mapped markers (21.6%) at which alleles did not segregate according to the expected Mendelian ratio. Out of the 214 markers, 158 SNPs (mostly on the LGIII, LGV, and LGVI) and 56 SNPs (on LGI, LGII, and LGVII) showed allelic segregation with a lower (< 3%) and higher (> 15%) frequency of E11 alleles, respectively, than the expected Mendelian allelic frequency of 12.5%.

**Table 1:** Pea populations and number of markers used in this study.

Collection or population	Number of pea lines	Number of markers used for genotyping						Reference
		Total	PsCam	Ps1	Ps0	Ps9	Other markers^a^	
Pea-Aphanomyces collection	172	10,824	9,549	1,214	21	35	5*	Desgroux et al. (2016)
RIL Baccara x PI180693 population	176	1,866	291	1,292	74	28	181**	Hamon et al. (2011; 2013)
RIL Baccara x 552 population	178	1,082	210	690	61	14	107**	Hamon et al. (2011; 2013)
RIL DSP x 90-2131 population	111	950	203	553	46	11	137**	Hamon et al. (2013)
RIL Puget x 90-2079 population	121	669	190	286	7	17	169**	Pilet-Nayel et al. (2002; 2005); Hamon et al. (2013); Lavaud et al. (submitted^1^)
AB Eden x E11 population	160	993	242	712	15	22	2*	This study
AB Eden x LISA population	178	478	317	143	8	9	1*	This study

a Other markers including (*) SNP markers and (**) markers from Pilet-Nayel et al. (2002; 2005) and Hamon et al. (2011; 2013).

Out of the 1,850 supplementary marker set used in this study, 1,832 markers were well projected from individual genetic maps onto the pea reference consensus genetic map (Tayeh et al., 2015), and contributed to densify the final consensus marker map DORA which comprises 16,647 markers and covers 801.2 cM Haldane. The consensus marker map DORA has an average marker density ranging from 19.1 to 22.1 markers/cM, depending on LGs, and includes seven gaps ranging from 1.0 to 5.7 cM between two contiguous markers (**Supplementary Table 2**).

### QTL mapping

**Figure 2:**
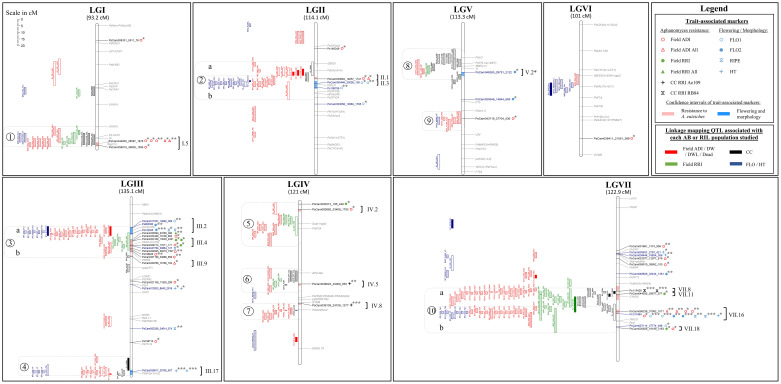
Comparative genetic mapping of markers and LD blocks identified by GWAS, and QTL detected by AB and RIL linkage analyses, for resistance to *A. euteiches*. LG names from I to VII and LG sizes in cM Haldane. (i) On the right of each LG: names of significant SNPs identified by GWAS in black and blue with, in red and blue shading in the LG bar, the confidence intervals around the significant SNPs (r^2^ > 0.7) for resistance and flowering/morphology, respectively; names and genomic positions of cloned pea genes in grey; on the right of each SNP significant marker, symbol(s) for the trait(s) associated markers as described in the legend, and significance of the marker-trait association (*p-value*: ‘***’ ≤ 1.E-12 < ‘**’ ≤ 1.E-6 < ‘*’); on the far right of each LG, names of the defined LD blocks according to Desgroux et al. (2016), with brackets gathering the markers attributed to the same LD block (r^2^ > 0.7). (ii) On the left of each LG: QTL detected by linkage mapping for the different variable types studied, as described in the legend; empty and filled rectangles for QTL from Eden x LISA and Eden x E11 AB populations, respectively; diagonal, horizontal, vertical, and crossed bar rectangles for QTL from Baccara x 552, Baccara x PI180693, DSP x 90-2131 and Puget x 90-2079 RIL populations, respectively; rectangle lengths as QTL confidence intervals (one LOD drop-off); on the far left of QTL, numbers from 1 to 10 surrounded by grey dotted rectangles, for consistent genetic regions of partial resistance to *A. euteiches*; sub-regions a and b delimited by grey dotted lines in regions 2, 3 and 10, to separate resistance QTL colocalizing or not with flowering or morphological QTL.

A total of 11, 13, 61, 34, 39, and 13 additive-effect QTL were detected for Aphanomyces root rot resistance in the Eden x E11, Eden x LISA, Baccara x PI180693, Baccara x 552, DSP x 90-2131, and Puget x 90-2079 populations, respectively. The characteristics and genetic localizations of these QTL are indicated in **Figure 2** and **Supplementary Table 3**.

In the Eden x E11 population, six genetic regions were significantly associated with resistance. Two of these regions were detected from several variables, including one region on LGIII close to the *Le* (internode length) locus in the *Ae-Ps3.2* QTL region (ADI and RB84, 6.4% < R² < 6.7%) and the other one on LGVII in the *Ae-Ps7.6* region (RRI, RB84 and Ae109, 9.1% < R² < 53.6%) with a major effect associated with the RB84 variable (R² = 53.6%). Four regions were detected from one or several ADI variable(s), on LGII in the *Ae-Ps2.2* region, on LGIII close to the *Hr* (flowering) locus in the *Ae-Ps3.1* region, on LGIV and on LGVII in the *Ae-Ps7.5* region (8.5% < R² < 22.3%). In the Eden x LISA population, six genetic regions were also significantly associated with resistance, including (i) the three regions detected from the Eden x E11 population on LGII, LGIII (close to *Le*) and LGVII, the one on LGII showing a consistent and higher effect (ADI and RB84 variables; 7.3% < R² < 37.6%) in contrast to that on LGVII (Ae109, R² = 7.6%) and (ii) three other minor-effect regions (R² < 9.5%), each detected with one or two variables, on LGI in the *Ae-Ps1.3* region, LGIII, and LGIV close to the *Ae-Ps4.1* region. In each population, allelic contribution to the resistance was brought by the resistant parent, E11 or LISA, at all the QTL detected but one showing minor and inconsistent effect. Four additive-effect QTL associated with early flowering were identified, including QTL co-localizing with resistance genetic regions detected on LGII in the *Ae-Ps2.2* region and LGIII close to the *Hr* or *Le* locus, in both populations. In the co-localizing region on LGII, the QTL detected for flowering showed a major additive-effect (46.8% < R² < 70.6%, depending on the AB population) and an epistatic effect (R^2 ^= 3.2%) with another QTL detected for flowering in the Eden x E11 population. In this region, resistance-increasing alleles from E11 or LISA contributed to late flowering.

A total of 50, 31, 37, and 13 resistance additive-effect QTL previously detected in Pilet-Nayel et al. (2002; 2005) and Hamon et al. (2011; 2013), were re-identified in the Baccara x PI180693, Baccara x 552, DSP x 90-2131, and Puget x 90-2079 RIL populations, respectively, using supplemented genotyping datasets and updated linkage analysis studies. In the Puget x 90-2079 population, no additional QTL was identified compared to those previously described in Pilet-Nayel et al. (2002; 2005). In the Baccara x PI180693, Baccara x 552 and DSP x 90-2131 populations, 11, three and two additional resistance QTL were detected, for Ae109, ADI, DW and RRI variables (3.9% < R² < 42.0%), ADI variables (6.5% < R² < 10.7), and Ae87 and Dead variables (R² = 8.3% and 42.2%, respectively), respectively. All additional QTL were detected in genetic regions previously associated with Aphanomyces root rot resistance on LGI (*Ae-Ps1.1*, *Ae-Ps1.2*), LGII (*Ae-Ps2.2*), LGIII (*Ae-Ps3.1*, *Ae-Ps3.2*), LGIV (*Ae-Ps4.5*), LGV (*Ae-Ps5.2*), and LGVII (*Ae-Ps7.5*, *Ae-Ps7.6*). In the Baccara x PI180693 and Baccara x 552 RIL populations, 8 and 4 additional QTL associated with flowering or height traits were identified, respectively, in genetic regions previously detected for these traits (on LGI in *Ae-Ps1.2*, LGII in *Ae-Ps2.1* and *Ae-Ps2.2*, LGIII in *Ae-Ps3.2*, LGVII in *Ae-Ps7.4* and *Ae-Ps7.6* regions) and in a new region on LGVI between the *Ae-Ps6.2* and *Ae-Ps6.4* QTL. Pairwise epistatic interactions associated with flowering traits (R² < 9.5%) were identified in the Baccara x PI180693 and DSP x 90-2131 RIL populations.

### Genome-wide association mapping

LD, structure and kinship data on the pea-Aphanomyces collection were updated using the genotyping dataset of 10,824 SNPs on 172 pea genotypes, presented in Desgroux et al. (2016). The LD decay value averaged 0.14 cM (**Supplementary Figure 2A**), which was close to the value (0.12 cM) presented in Desgroux et al. (2016). Ten genetic sub-populations were identified in the collection, according to the optimal likelihood value of K computed by ADMIXTURE (**Supplementary Figure 2B**). These results completed the PCA analysis conducted in Desgroux et al. (2016), which integrated into GWAS the structure captured by the three first PCA axis. Kinship analysis revealed IBD relatedness coefficients ranging from -0.34 to 1.12 among individuals with an estimated average of -0.006 (**Supplementary Figure 2C**). The Ward’s clustering computed on kinship IBD matrix showed consistent clusters with those described in Desgroux et al. (2016).

Updated GWAS in the collection identified a total of 29 resistance-associated SNPs distributed over the seven LGs, using MLMM with a maximum number of cofactors of 5 and *p-value* < 3.6E-05 (**Figure 2** and **Supplementary Table 4**). This number of significant SNPs was lower compared to the 56 resistance-associated markers described in Desgroux et al. (2016), using MLMM with a maximum number of cofactors of 10 and *p-value <* 2.5E-05. Significant markers were associated with 22 variables, for which a total of 41 marker-trait associations including 14 previously identified (Desgroux et al., 2016), were significantly identified as cofactors in the MLMM model (4.61E-28 *< p-value <* 3.57E-05). Zero to four markers were detected as cofactors for each variable, explaining a total of 0 to 68% of the phenotypic variation. A total of 17 markers significantly associated (9.27E-25 < *p-value* < 2.86E-05) with flowering or morphological variables, and 28 marker-trait associations including five previously detected (Desgroux et al., 2016), were identified on LGII, LGIII, LGV, and LGVII (**Figure 2** and **Supplementary Table 4**).

### Consistent genetic regions of resistance

A total of ten consistent genetic regions of partial resistance to *A. euteiches*, numbered from 1 to 10, were identified according to the colocalization of at least four partial resistance QTL and/or resistance-associated SNPs identified by RIL and/or AB linkage analyses and/or GWAS, respectively (**Figure 2**, **Table 2** and **Supplementary Table 5**). On LGIV, the intervals of two genetic regions named 5 and 7 were slightly extended to include significant resistance QTL or resistance-associated SNPs detected by GWAS flanking the positions of the four co-located QTL/SNPs of each region. The size of each consistent genetic region ranged from 12.1 to 34.0 cM (genetic regions n°7 and n°10, respectively), depending on the region. The number of resistance QTL and resistance-associated GWAS-SNPs comprised in each consistent genetic region ranged from 4 to 55 and 0 to 8, respectively (**Figure 2**). The ten consistent regions cover those of the seven main consistent QTL and three additional less consistent QTL (*Ae-Ps3.2*, *Ae-Ps4.3* and *Ae-Ps5.2*), previously reported (Hamon et al., 2013). Most of the consistent genetic regions comprised resistance QTL and GWAS-SNPs associated with field and controlled conditions variables, except for the fourth and eighth regions including no resistance-associated marker. Both major resistance *Ae-Ps4.5* and *Ae-Ps7.6* QTL were re-identified in QTL mapping and GWAS with a high proportion of phenotypic variation explained by each individual QTL (R^2 ^= 88.9% and 74.8%, respectively) and a high significance level of resistance-associated GWAS-SNP (*p-value* = 4.6E-28 and 3.6E-16, respectively) (**Table 2**). Each consistent genetic region colocalized with at least one flowering/morphological QTL or -associated GWAS-SNP, except for genetic regions identified on LGIV (**Supplementary Table 5**). The *Ae-Ps2.2*, *Ae-Ps3.1* and *Ae-Ps7.6* regions were split into two genetic sub-regions, separating partial resistance QTL colocalizing with flowering and morphological QTL from other partial resistance QTL.

**Table 2:** Consistent genetic regions, including QTL, SNPs and LD blocks, contributing to partial resistance to *A. euteiches* in pea.

					Linkage analysis in AB and RIL populations						GWAS in the pea-Aphanomyces collection				
LG	Consistent genomic region^a^	Genetic subregion^b^	Position interval [minimum ; maximum]^c^	Genes^d^	*Ae-Psxxx QTL* ^e^	Nb of population	Nb of QTL	Nb of QTL associated variable	R^2^ mean ± sd	Range of R^2^ value	LD block	Nb of GWAS-SNP	Nb of GWAS-SNP associated variable	*p-value* mean ± sd	Range of *p-value*
**I**	**1**	**-**	[76.6 ; 91.8]	**Af / SGR**	*Ae-Ps1.2*	3	14	7 ADI + 4 RRI + 1 Ae106_TDL + 1 SP7_TDL + 1 Ae106_TWL	11.5 ± 3.8	[5.7 ; 16.6]	I.5	6	6 ADI	1.3E-05 ± 1.3E-05	[3.9E-11 ; 3.0E-05]
**II**	**2**	**a**	[29.1 ; 39.7]	**A**	*Ae-Ps2.2a*	5	10	8 ADI + 2 RB84	18.7 ± 10.3	[7.2 ; 37.6]	–	–	–	–	–
		**b**	[39.9 ; 57.9]		*Ae-Ps2.2b*	4	7	6 ADI + 1 RB84	11.3 ± 4.7	[7.2 ; 20.4]	II.1	2	2 ADI	1.6E-05 ± 1.3E-05	[6.6E-06 ; 2.5E-05]
**III**	**3**	**a**	[21.5 ; 29.5]	**HR**	*Ae-Ps3.1a*	2	14	13 ADI + 1 RRI	17.6 ± 7.0	[7.6 ; 29.4]	–	–	–	–	–
		**b**	[29.5 ; 45.4]	**-**	*Ae-Ps3.1b*	2	8	3 ADI + 4 RRI+ 1 RB84	10.5 ± 4.1	[7.6 ; 19.4]	III.4 / III.9	10	6 ADI + 4 RRI	5.8E-06 ± 9.2E-06	[4.0E-09 ; 2.6E-05]
	**4**	**-**	[120.4 ; 135.1]	**LE**	*Ae-Ps3.2*	3	11	7 ADI + 1 DW + 1 Ae109 + 2 RB84	11.9 ± 6.6	[5.6 ; 28.5]	–	–	–	–	–
**IV**	**5**	**-**	[0.7 ; 33.4]	**-**	*Ae-Ps4.1*	3	10	7 ADI + 3 RRI	11.5 ± 7	[5.8 ; 26.1]	IV.2	2	ADI + 1 RRI	2.7E-05 ± 1.3E-05	[1.7E-05 ; 3.6E-05]
	**6**	**-**	[50.0 ; 67.7]	**-**	*Ae-Ps4.3*	3	7	2 ADI + 2 RRI + 1 Ae87 + 1 SP7_TDL + 1 SP7_TWL	14.9 ± 14.7	[3.4 ; 46.8]	IV.5	1	1 Ae109	7.8E-07	–
	**7**	**-**	[76.1 ; 88.2]	**-**	*Ae-Ps4.5*	2	7	5 ADI + 1 DWL + 1 Ae109	31.3 ± 30	[3.9 ; 88.9]	IV.8	1	1 Ae109	4.6E-28	–
**V**	**8**	**-**	[19.0 ; 42.2]	**r**	*Ae-Ps5.1*	3	11	2 ADI + 1 RRI + 2 Ae109 + 1 Ae106 + 1 Ae85 + 1 Ae78 + 3 RB84	15.8 ± 10.5	[2.2 ; 37.2]	–	–	–	–	–
	**9**	**-**	[66.5 ; 80.8]	**-**	*Ae-Ps5.2*	2	4	4 ADI	12.1 ± 5.8	[6.5 ; 20.3]	–	1	1 ADI	4.1E-06	–
**VII**	**10**	**a**	[68.3 ; 81.6]	**-**	*Ae-Ps7.6a*	5	39	17 ADI + 1 Dead + 10 RRI + 4 Ae109 + 1 Ae85 + 1 Ae78 + 1 Ae106 + 1 Ae87 + 3 RB84	27.0 ± 19.5	[7.5 ; 74.8]	VII.8 / VII.11	3	1 RRI + 2 RB84	7.8E-06 ± 1.4E-05	[3.6E-16 ; 2.4E-05]
		**b**	[81.6 ; 102.3]	**-**	*Ae-Ps7.6b*	5	25	13 ADI + 1 DW + 7 RRI + 3 Ae109 + 1 Ae87	16.5 ± 10.4	[6.5 ; 44.0]	VII.16 / VII.18	7	6 ADI + 1 RRI	5.8E-06 ± 8.1E-06	[1.6E-07 ; 2.0E-05]

^a^Consistent genetic regions and ^b^sub-regions, as described in **Figure 2**, with their ^c^intervals in cM Haldane located on the consensus marker map DORA.

^d^Morphological genes present in consistent genetic regions.

^e^Ae-Psxxx QTL from Hamon et al. (2013) repositioned on the consensus marker map DORA according to updated linkage analyses in RIL populations.

R²: percentage of phenotypic variation explained by a QTL. LD blocks as described in **Figure 2**. Variable codes as described in **Supplementary Table 1**, with field variables on aerial and root plant part written in red and green, respectively, and controlled conditions variables in dark.

### LD blocks

A total of 11 resistance LD blocks, covering 0.1 to 8.1 cM, were identified around significant resistance-associated GWAS-SNPs comprised in the ten consistent genetic regions. Each LD block included 1 to 22 markers in LD (r^2^ > 0.7) with each significant marker (**Figure 2** and **Supplementary Table 6**). All disease resistance LD blocks comprised at least one common SNP with the markers composing the LD blocks detected in Desgroux et al. (2016), and were thus named as presented in the previous study. Out of the 11 LD blocks, (i) two were common to field ADI and RRI traits (III.4 and VII.18 in *Ae-Ps3.1* and *Ae-Ps7.6* regions, respectively), (ii) five and one were specific to field ADI (I.5, II.1, III.9, IV.2, and VII.16 in *Ae-Ps1.2*, *Ae-Ps2.2*, *Ae-Ps3.1*, *Ae-Ps4.1*, and *Ae-Ps7.6* regions, respectively) and RRI (VII.11 in the *Ae-Ps7.6* region) variables, respectively, (iii) and two and one were specific to the Ae109 (IV.5 and IV.8 in *Ae-Ps4.3* and *Ae-Ps4.5* regions, respectively) and RB84 (VII.8 in the *Ae-Ps7.6* region) strains evaluated in controlled conditions, respectively.

Five LD blocks, which intervals ranged from 3.0 to 5.9 cM, were defined around significant flowering and morphological-associated SNPs identified in five consistent genetic regions. Among the five LD blocks, two and one LD blocks were specific to flowering (II.3 and V.2* in *Ae-Ps2.2* and *Ae-Ps5.1* regions, respectively) and height (III.17 in the *Ae-Ps3.2* region) variables, respectively. Two were common to flowering, height, or ripening variables (III.2 and VII.16 in *Ae-P3.1* and *Ae-Ps7.6* regions, respectively). Flowering and morphological LD blocks were named according to Desgroux et al. (2016), except for the LD block V.2 (renamed V.2*) which was associated with a different variable compared to the one identified in the previous study. Only the LD block VII.16 associated with flowering and morphological variables was common to *A. euteiches* resistance. At this LD block, opposite allelic effects were found at significant resistance and flowering/morphological-associated SNPs, which suggest that resistance-enhancing alleles contributed to higher plants and later flowering and ripening (**Figure 2** and **Supplementary Tables 4**, **6**).

### Marker haplotypes

**Figure 3:**
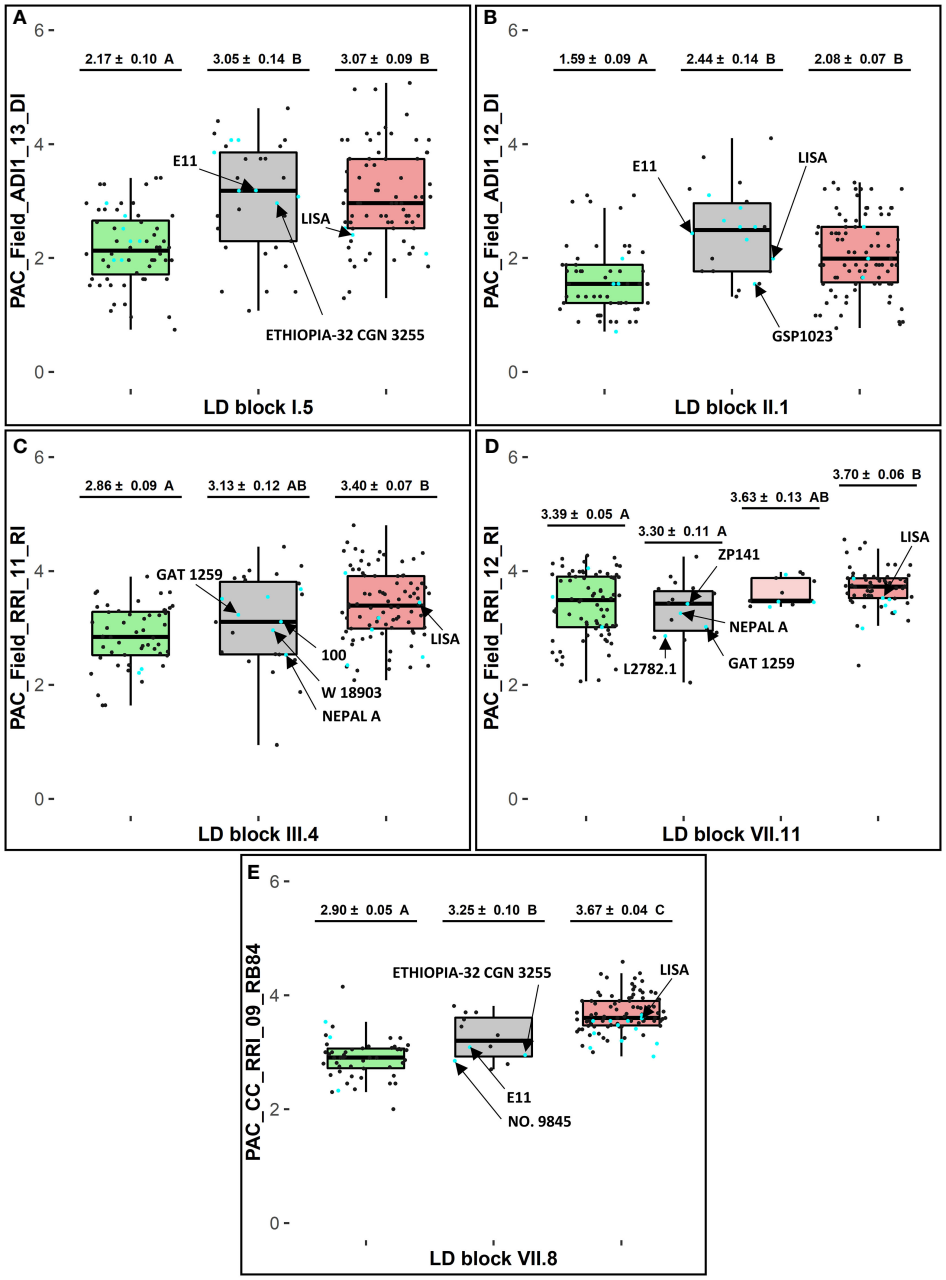
Haplotype analysis at selected LD blocks in consistent genetic regions (1, 2b, 3b and 10a) for selected variables of resistance to *A euteiches*. At each LD block **(A)** I.5, **(B)** II.1, **(C)** III.4, **(D)** VII.11, and **(E)** VII.8: adjusted EMMs phenotypic scores ± standard deviation and Tukey-HSD mean comparison groups (α = 5%), for each favorable (in green), rare (in grey), and unfavorable (in red) haplotype box with n = 1 haplotype in each green and red box and n ≥ 2 haplotypes in each grey box; cyan and dark dots showing phenotypic EMMs values of pea lines belonging or not, respectively, to the 20 new sources of resistance defined in the pea-Aphanomyces collection; arrows pointing values for E11 and LISA, or for new sources of resistance carrying rare haplotypes which showed lower values than the average ones of lines sharing the unfavorable haplotype.

At each of the 11 LD blocks defined around significant resistance-associated GWAS-SNPs, three to twenty-one marker haplotypes showing different genotyping profiles were identified, depending on the LD block (**Supplementary Table 6**). At each resistance LD block, one favorable haplotype and one unfavorable haplotype, well-represented in the pea-Aphanomyces collection (frequency > 5%), was identified by mean comparison of phenotypic EMMs between each haplotype group (α = 5%). The pea lines AeD99QU-04-4-6-1, AeD99QU-04-15-8-1, and AeD99OSW-49-5-7 cumulated the highest number of favorable haplotypes (n = 8) at the 11 resistance LD blocks and displayed a high level of disease resistance in the pea-Aphanomyces collection (**Supplementary Table 7**). At each LD block, one to 19 rare haplotypes (frequency ≤ 5%) were detected, depending on the LD block. At five of the 11 LD blocks showing the most significant resistance-associated GWAS-SNPs, the group of pea lines bringing together several rare haplotypes (number of rare haplotypes ≥ 2, cumulated frequencies > 5%) presented a mean resistance level significantly similar (blocks VII.11 and III.4) or lower (blocks I.5, II.1 and VII.8) than the group of pea lines sharing the favorable haplotype (α = 5%) (**Figure 3**).

Among 14 pea accessions classified as new sources of resistance in the collection and found to carry rare haplotypes in this study, several lines exhibited a higher level of resistance compared to the average resistance level of lines sharing the favorable haplotype. These lines are especially NEPAL A at LD block III.4, and NEPAL A, GAT1259, L2782.1 at LD block VII.11. The AB parental line E11, carrying a rare haplotype at LD block VII.8 in the *Ae-Ps7.6* region, had a lower disease severity in inoculated conditions with the RB84 strain than the group of pea lines sharing the unfavorable haplotype at this LD block (**Figure 3**). The AB parental line LISA, carrying unfavorable haplotypes at eight of the 11 LD blocks and a rare haplotype at LD block II.1 in the *Ae-Ps2.2* region, displayed lower susceptibility in the field (**Supplementary Table 7**). Favorable and unfavorable haplotypes were also detected at three of the five LD blocks identified around significant flowering and morphological-associated SNPs (**Supplementary Tables 6**, **7**).

## Discussion

This study aimed to identify novel QTL and alleles, as well as QTL-closely linked SNPs, for resistance to Aphanomyces root rot in pea, a complex genetically inherited trait. Indeed, diversifying and efficiently pyramiding resistance QTL and alleles in breeding will be necessary to develop pea varieties with high and durable levels of resistance to Aphanomyces root rot, which remains challenging. This work describes the first AB-QTL mapping approach to investigate the polygenic control of Aphanomyces root rot resistance in new sources of resistance. It describes an update of previous QTL/GWAS studies by incorporating common SNP markers with the AB-QTL mapping approach. This integration was necessary for analyzing the novelty of QTL identified in AB populations based on their genetic locations.

### AB-QTL mapping in two new sources of resistance confirmed previous resistance QTL

AB mapping is a suitable approach both to detect and advance introgression of exotic alleles originating from germplasms genetically distant from elite varieties. In addition, QTL mapping in AB populations makes it possible to identify rare QTL or alleles in an agronomic background (Tanksley and Nelson, 1996), which would not be detectable in GWAS approach. Among the 20 new sources of resistance selected from the extensive screening program of Aphanomyces resistance accessions previously conducted by Pilet-Nayel et al. (2007), E11 and LISA were identified as two new pea germplasm lines partially resistant to *A. euteiches.* These two lines showed rare marker haplotypes at consistent resistance loci in the pea Aphanomyces collection (Desgroux et al., 2016), which were mostly different from those derived from the reference parents PI180693, 552, 90-2131 and 90-2079. We thus produced AB populations using E11 and LISA as partially resistant parents, to identify new QTL for resistance to *A. euteiches* and advance their introduction into an agronomic genetic background (Eden) previously used for NIL creation at Aphanomyces resistance QTL (Lavaud et al., 2015).

In this study, QTL mapping detected 11 and 13 additive-effect QTL associated with *A. euteiches* resistance in the Eden x E11 and Eden x LISA AB populations, respectively. These QTL were located in the *Ae-Ps1.3*, *Ae-Ps2.2*, *Ae-Ps3.1*, *Ae-Ps3.2*, *Ae-Ps4.1*, *Ae-Ps7.5* and *Ae-Ps7.6* regions, previously reported (Hamon et al., 2011; Hamon et al., 2013; Desgroux et al., 2016). Highest effects of resistance QTL were identified in the *Ae-Ps7.6* region (8.5% < R^2^ < 53.6%) from the Eden x E11 population and in the *Ae-Ps2.2* region (7.3% < R^2^ < 37.6%) from both AB populations. At QTL *Ae-Ps7.6*, we can hypothesize that the resistance allele with major effect identified in E11 could be identical to those identified with major effects in PI180693 and 90-2131. However, to our knowledge, no pedigree data from the parental line E11 is available to highlight potential genetic relationship with PI180693 and 90-2131. Genome sequencing of the three genotypes at QTL *Ae-Ps7.6* would be helpful to address this issue. At QTL *Ae-Ps2.2*, we can presume that the resistance alleles in E11 and LISA are closely-linked or correspond to late flowering alleles, since co-localizations were observed between resistance and flowering QTL with opposite allelic effects as reported in Hamon et al. (2013). Pleiotropy or linkage between genes controlling plant disease resistance and undesired development traits, *e.g.* late-flowering, were commonly reported in the literature (Poland et al., 2009). In addition, resistance alleles in the *Ae-Ps2.2* region cosegregated with colored flower alleles in pea lines of the two AB populations, suggesting pleiotropy or genetic linkage between the resistance QTL and the *A* morphological gene controlling anthocyanin production (*i.e.* the PsbHLH gene). Updated GWAS refined the localization of the resistance LD block II.1 in the *Ae-Ps2.2* region to a genetic position close to but different from the *A* locus as previously highlighted by Desgroux et al. (2016), which suggests the possibility to break the negative correlation between resistance and colored flowers in pea.

In the Eden x LISA and Eden x E11 populations, AB lines showing highest levels of partial resistance to *A. euteiches* carried resistance alleles at QTL *Ae-Ps2.2* or *Ae-Ps7.6*, individually (lines EL.61, EL.143 and lines E11.87, E11.133, respectively) or in combination with resistance alleles at one or two additional minor-effect QTL (*Ae-Ps2.2* and *Ae-Ps1.3*, *Ae-Ps3.2* or *Ae-Ps7.6* in lines EL.65, EL.186; *Ae-Ps7.6* and *Ae-Ps2.2*, *Ae-Ps3.1*, *Ae-Ps3.2* or *Ae-Ps7.5* in lines E11.9, E11.42, E11.68, E11.79). By advancing backcross generations to the elite background, the frequency of undesirable alleles from the unadapted donor line is reduced in AB populations, favoring the transfer of valuable QTL into established elite inbred lines (Tanksley and Nelson, 1996). In the two AB populations studied, allele frequencies skew towards the recurrent breeding parent Eden (frequency > 87.6%), favoring the future development of NILs by further backcrossing selected AB lines to the agronomic recipient parent Eden.

### Integrated linkage and genome-wide association mapping identified ten consistent genetic regions and closely-linked SNP markers associated with resistance

Comparative mapping is a valuable approach to compare QTL identified from different populations and genetic analysis methods, thus providing insight into their organization and diversity across the genome. This approach is based on the use of common “bridge” markers between genetic studies and mapping populations, in sufficiently high density to precisely compare QTL positions between genetic maps. So far, the comparative mapping of Aphanomyces root rot resistance QTL detected by linkage mapping in the RIL populations and by GWAS in the pea-Aphanomyces collection has only been performed previously based on the projection of 144 common markers onto a consensus map (Desgroux et al., 2016). In this study, we used 1,850 markers publicly available (Duarte et al., 2014; Tayeh et al., 2015; Boutet et al., 2016), including 462 SNPs anchored to the pea genome (Kreplak et al., 2019), as bridge markers between AB and RIL studies and GWAS to accurately compare the QTL positions re-detected in the different studies on the consensus marker map DORA. These SNP markers also made possible to localize the most stable major-effect QTL *Ae_MRCD1_Ps-4.1/2* recently reported in Wu et al. (2021) in the region of QTL *Ae-Ps4.5*.

In addition, the QTL detection methods in RIL populations (Pilet-Nayel et al., 2002; Pilet-Nayel et al., 2005; Hamon et al., 2011; Hamon et al., 2013) and the pea-Aphanomyces collection (Desgroux et al., 2016) have been revisited with more stringency, allowing more robust QTL re-detection. In QTL mapping from the four RIL populations, higher minimum LOD score thresholds (2.8 < LOD < 4.8) were estimated in updated composite interval mapping analyses compared to the ones (2.8 < LOD < 2.9) computed in previous studies. This resulted in the detection of more consistent QTL and fewer putative false positives, confirming the seven main *Ae-Ps* meta-QTL and invalidating 15 to 42 minor-effect resistance QTL (R^2^ < 27.2%) previously detected, depending on the population. In addition, in the Baccara x PI180693 population, updated analysis identified mismatches between phenotyping and genotyping datasets from Hamon et al. (2011), showing three spurious resistance QTL associated with resistance to the Ae109 and/or RB84 strains that were previously detected on LGII, LGVI, and LGVII (8.7% < R^2^ < 49.4%). Updated QTL mapping taking into account these corrections, showed colocalization in the *Ae-Ps7.6* region between PI180693 and 90-2131 alleles contributing to resistance to the RB84 strain, in accordance with the pedigree relationship between these two resistant germplasms (Kraft, 1992). In this study, we observed that major QTL identified in controlled conditions accounted for a higher percentage of phenotypic variation compared to the QTL detected in field environments in AB and RIL populations. This discrepancy in variance could be attributed to the higher level of control over environmental factors in controlled conditions, such as temperature, humidity, light, and nutrient availability, resulting most often in higher heritability values.

In the updated GWAS of the pea-Aphanomyces collection, we confirmed 29 resistance-associated SNPs out of the 56 resistance markers previously reported. This lower number of associated SNPs detected was mostly explained in the MLMM model by (i) a minimum number of cofactors associated with a single variable set at 5 instead of 10 in Desgroux et al. (2016), (ii) a higher *p-value* threshold to declare significant cofactors (*p-value* = 3.6E-05), and (iii) a larger percentage of phenotypic variation explained by the structure matrix computed with ADMIXTURE. These new parameters used for GWAS resulted in the reduction of the number of low-effect loci (1.0E-07 < *p-value* < 3.6E-05) detected in this study compared to Desgroux et al. (2016).

Integrating bi-parental linkage mapping and GWAS approaches allows to take advantage of both methods while limiting the drawbacks of each of them, which improves the identification of consistent candidate loci in plants (He et al., 2017; Guo et al., 2019; Zhao et al., 2022). In our study, comparative mapping of Aphanomyces resistance QTL and resistance-associated GWAS-SNPs identified 10 consistent genetic regions on the consensus marker map DORA, each of them being identified from at least three of the seven populations studied. It refined, with a high resolution, the positions of the main *Ae-Ps* QTL previously described in Hamon et al. (2011; 2013) and identified novel SNPs flanking the intervals of these regions (12.1 cM ≤ interval ≤ 34.0 cM). A total of 11 LD blocks associated with Aphanomyces root rot resistance were detected in 10 consistent genetic regions, including at least one common SNP with the markers composing the resistance LD blocks previously identified by Desgroux et al. (2016). Three of the 10 consistent genetic regions (2.a, 3.b, and 10.a) associated with partial resistance QTL could be divided into sub-regions, separating resistance QTL from flowering or morphological QTL, which suggests the possibility of breaking undesirable correlations between traits in pea breeding.

### Rare and favorable marker haplotypes were identified for QTL pyramiding and diversification in breeding for pea resistant varieties

Haplotype analysis is a powerful tool to reveal rare alleles in LD with molecular markers (Bhat et al., 2021), especially because rare causal variants often determine extreme phenotypes (Wray et al., 2013). In plant collections, new germplasms provide a useful genetic diversity to develop durable disease-resistant cultivars (Thudi et al., 2021). In our study, the new source of resistance E11 showed five rare haplotypes in the *Ae-Ps1.2*, *Ae-Ps2.2*, *Ae-Ps4.5*, and *Ae-Ps7.6* genetic regions and a high rate of missing haplotypes (n = 5/11). Especially, the high level of resistance to RB84 strain was explained in E11 by the rare haplotype VII.8.d. Although LISA combined few haplotypes significantly detected as favorable (n = 2/11), this new source of resistance displayed reduced aerial symptoms in infested field conditions. Partial resistance to *A. euteiches* in LISA was especially associated with the rare haplotype II.1.m identified in the *Ae-Ps2.2* genetic region and the haplotype effect was confirmed by linkage analysis in the Eden x LISA AB population.

Nine other new sources of resistance, clustered into the same kinship group as the winter pea varieties, showed a high number of rare haplotypes (1 ≤ n ≤ 7) at 11 resistance LD blocks. In particular, rare haplotypes in NEPAL A (III.4.e and VII.11.l), NO. 9845 (VII.8.f), GAT 1259 and L2782.1 (VII.11.i and VII.11.k, respectively) were associated with a higher level of resistance than the average level of the pea lines in the collection carrying favorable haplotypes at these LD blocks. Beji et al. (2020) recently revealed colocalization in the *Ae-Ps1.1* and *Ae-Ps7.6* regions between LD blocks detected in GWAS for frost tolerance, and QTL and LD blocks associated with Aphanomyces root rot resistance identified in previous studies (Hamon et al., 2011; Desgroux et al., 2016). This may suggest that rare alleles in this region may contribute to resistance to multiple stresses, which is a major breeding goal in pea (Burstin et al., 2021), and that it may be possible to breed favorably for Aphanomyces resistance and frost tolerance in future pea breeding programs.

Haplotype analysis has been highly relevant to reveal combinations of favorable haplotypes to be used in marker-assisted selection. Pyramiding of QTL for resistance to pathogens is a promising approach to increase levels of resistance and limit QTL erosion in breeding lines (Pilet-Nayel et al., 2017). Lavaud et al. (2016) reported that the combination of resistance alleles at two or three of the main resistance QTL, including the major-effect *Ae-Ps7.6* QTL, increased partial resistance to *A. euteiches* in pea NILs. Additionally, the combination of the *Ae-Ps7.6* QTL with other QTL was recently suggested to preserve the durability of the major QTL, since aggressive isolates on NILs carrying *Ae-Ps7.6* were found in *A. euteiches* natural populations (Quillévéré-Hamard et al., 2021). In our study, haplotype analysis at 11 resistance LD blocks in ten consistent genetic regions confirmed that lines showing the highest level of partial resistance to *A. euteiches* carried mostly favorable haplotypes (3 < n < 7) in combination with the resistance haplotype VII.8.a located in the major-effect *Ae-Ps7.6* genetic region. The most resistant lines in the collection, derived from the AeD99 phenotypic recurrent breeding program, carried a higher number of favorable haplotypes (n = 6) originating from the combination of the three reference resistant parents 90–2131, PI180693 and 552 (Desgroux et al., 2016). However, the best AeD99 breeding lines cumulated also two to three unfavorable haplotypes at three LD blocks (II.3, V.2*, and VII.16) associated with flowering and morphological traits. In particular, negative linkages between haplotypes associated with resistance and developmental traits were identified at the LD block VII.16. The combination of major-effect QTL with multiple small-effect QTL, coupled with the breakage of negative linkages, is a promising approach to enhance resistance levels against *A. euteiches* in future pea varieties. Recent registrations of French tolerant varieties carrying several Aphanomyces resistance QTL further support the effectiveness of this strategy (Moussart, 2022).

## Conclusion

This study provides an overview of the diversity of QTL and haplotypes that significantly contribute to Aphanomyces root rot resistance in pea, by integrating AB-, RIL-linkage mapping and GWAS data using 1,850 common SNP markers. Most of the previously identified resistance QTL were confirmed and mapped onto a consensus marker map. No new consistent resistance QTL were identified in both Eden x E11 and Eden x LISA AB populations. However, ten consistent genetic regions comprising resistance QTL with closely linked new SNPs, as well as favorable haplotypes in these regions, were identified and appear to be good choices for future resistance allele pyramiding in marker-assisted selection strategies. New relevant rare haplotypes identified in new sources of resistance and negative associations between resistance and undesirable alleles in targeted regions will remain to be explored in future pea breeding programs. Another major challenge will consist in identifying and validating candidate genes underlying Aphanomyces resistance QTL in pea.

## Data availability statement

The original contributions presented in the study are included in the article/**Supplementary Material**. Further inquiries can be directed to the corresponding author.

## Author contributions

TL participated in phenotypic data acquisition, managed all the statistical and genetic analyses, and drafted the manuscript. GB and CL generated genotypic data and participated in updated linkage analyses in RIL populations. AL, J-PR, PV, IG, HM, ALR, and CL contributed to implementing experimental assays and evaluating plant disease resistance and development traits. CR-K and PD contributed to the setting up and implementation of this study. AS contributed to the drafting of the manuscript. M-LP-N and CL coordinated the overall study and the manuscript drafting. All authors read and approved the final manuscript.

## Footnotes

1 Lavaud, C., Lesné, A., Leprévost, T., and Pilet-Nayel, M.-L. (submitted). Fine mapping of *Ae-Ps4.5*, a major locus for resistance to pathotype III of *Aphanomyces euteiches* in pea. Theor. Appl. Genet.

## Supplementary material

Supplementary Figure 1 Frequency distribution of EMMs obtained for *A. euteiches* resistance and flowering variables in the **(A)** Eden x E11 and **(B)** Eden x LISA AB populations. Scoring variables are coded as presented in **Supplementary Table 1**. Reference susceptible Eden and partially resistant E11 and LISA parents are indicated in red and green, respectively. n: total number of pea lines assessed; m: mean ± standard deviation; H^2^: mean-based heritability.

Supplementary Figure 2
**(A)** LD decay in the pea-Aphanomyces collection. Colored curves represent the estimated LD decay for each LG. Dashed vertical lines represent the LD threshold (maximum r^2^/2) and arrows the LD decay rate, as the estimated genetic distance (cM) to reach this LD threshold on each LG. **(B)** Population structure in the pea-Aphanomyces collection for 10 subgroups (Q). Each colored horizontal line of individual accession shows the ancestral fraction that was assigned proportionally to the estimated clusters. **(C)** Ward’s clustered heatmap of the kinship matrix of the pea-Aphanomyces collection. The color gradient represents the degree of relationship between two lines. Pea lines are gathered in 14 subgroups described in the legend.

Supplementary Table 1 Phenotypic data obtained for resistance to *A. euteiches*, flowering and morphological traits in the pea RIL and AB populations. 1^st^ to 4^th^ sheets: adjusted mean datasets for 33, 21, 31, and 12 variables previously evaluated in the Baccara x PI180693, Baccara x 552, DSP x 90-2131 and Puget x 90-2079 RIL populations, respectively. Data associated with Height (Baccara x PI180693 and Baccara x 552 RILs) and Dead (DSP x 90-2131 RILs) variables were not presented in Hamon et al. (2013). 5^th^ to 6^th^ sheets: EMMs for 9 variables evaluated in the Eden x E11 and Eden x LISA AB populations, respectively. 7^th^ sheet: description of resistance, flowering, and morphological variables presented in these phenotyping datasets. N.B. The adjusted means of phenotypic data in the pea-Aphanomyces collection can be found in the Additional file 16 of Desgroux et al. (2016).

Supplementary Table 2 Consensus marker map and genotyping data used in this study. 1^st^ sheet: consensus marker map DORA including 16,647 markers used to genotype one or several of the seven populations used in this study, synonymous marker names from Duarte et al. (2014) and Tayeh et al. (2015) are indicated in the second column; Genotyping matrices from: (2^nd^ sheet) the pea-Aphanomyces collection (10,824 filtered markers), (3^rd^ to 6^th^ sheets) RIL populations derived from the crosses Baccara x PI180693, Baccara x 552, DSP x 90-2131, and Puget x 90-2079, respectively (1,866, 1,082, 950, and 669 filtered markers, respectively), and (7^th^ to 8^th^ sheets) AB populations derived from the crosses Eden x E11 and Eden x LISA, respectively (993 and 478 filtered markers, respectively). 9^th^ sheet: coding information of reference parental alleles presented in previous genotyping datasets.

Supplementary Table 3 QTL detection results updated from previous phenotypic data in the Baccara x PI180693, Baccara x 552, DSP x 90-2131, Puget x 90-2079 RIL populations, and obtained in this study in the Eden x E11 and Eden x LISA AB populations, for resistance to *A. euteiches*, flowering, and morphological traits in controlled and field conditions. QTL are ordered by position in cM Haldane on the linkage group. ^a^ Scoring traits are coded as presented in the **Supplementary Table 1**. ^b^ Marker from the LOD score peak of the QTL (“-” attributed if no marker is located at the computed LOD score peak). ^c^ positions of epistatic QTL (interactions are shown as “LG of the first QTL@Position of the first QTL: LG of the second QTL@Position of the second QTL). ^d,e^ Mean effects of pea lines carrying allele from the susceptible parent (Baccara, DSP, Puget, or Eden) or the partially resistant parent (PI180693, 552, 90-2131, 90-2079, E11, or LISA) at the peak marker of the QTL. H²: broad-sense heritability value collected from previous genetic studies conducted on RIL populations and computed based on data obtained from two new AB populations in this study. LOD: log of likelihood ratio peak value at the QTL position for each variable. R²: percentage of phenotypic variation explained by a QTL.

Supplementary Table 4 GWAS detection results updated from previous phenotypic data in the pea-Aphanomyces collection for resistance to *A. euteiches*, flowering, and morphological traits. 1^st^ sheet: ^a^ genetic position (cM Haldane) of significant marker detected by GWAS on the DORA consensus marker map. ^b^ Variable name as described in the third table sheet. 2^nd^ sheet: partition of phenotypic variance for each variable. 3^rd^ sheet: description of resistance, flowering, and morphological variables evaluated in the pea-Aphanomyces collection.

Supplementary Table 5 Consistent genetic regions associated with partial resistance to *A. euteiches*, flowering, and morphology in pea. The **Supplementary Table 5** is an expanded version of the **Table 2**, including information on QTL and significant SNPs associated with Aphanomyces resistance, flowering and morphology variables, detected in the pea-Aphanomyces collection, and RIL and AB populations. LD blocks associated with resistance to *A. euteiches* and flowering or morphological traits are indicated in dark and blue, respectively. Variable codes as described in **Supplementary Tables 1** and **4**, with field variables on aerial and root plant part written in red and green, respectively, controlled conditions variables in dark, and flowering and morphology variables in blue.

Supplementary Table 6 Markers and haplotypes in the detected LD blocks. For each LD block: (1^st^ column) repositioned *Ae-Psxxx* QTL regions comprising LD blocks. (2^nd^ column) LD block number, as shown in **Figure 2**. (3^rd^ and 4^th^ columns) LG and genetic position of significant SNPs in the LD block on the DORA consensus marker map; (5^th^ column) SNPs significantly detected by GWAS in the LD block; (6^th^ column) variable coded as described in **Supplementary Table 4** from which each significant SNP was detected; (7^th^ column) *p-value* of each significant SNP (*p-value* < 3.6E-05); (following lines and columns) marker ID and genetic position on the DORA consensus marker map for each marker in the LD block, pairwise LD (r²) values between each marker defined in the LD block and markers detected by GWAS (detected markers in bold font and their markers in LD in plain font, “-” indicates r² values ≤ 0.7 between markers); haplotype letter, number and percentage of pea lines from the pea Aphanomyces collection sharing the haplotype; mean phenotypic values ± standard error and significantly different means (Tukey-HSD, α = 5%) for each haplotype carried by more than 5% of lines. Favorable and unfavorable haplotypes are shown in green and orange, respectively. Haplotypes carried by 5% or less than 5% of the pea lines in the pea Aphanomyces collection are considered as rare.

Supplementary Table 7 Marker haplotype description in the pea-Aphanomyces collection. ^a^ Names of the lines from the pea-Aphanomyces collection, as described in Desgroux et al. (2016); **Lines not retained in GWAS based on markers quality. Haplotype content (haplotype names in small letters) of each pea line in the collection at: ^b^ 11 consistent LD blocks associated with resistance to *A. euteiches* and ^e^ three consistent LD blocks associated with flowering or morphological traits, comprised in repositioned *Ae-Psxxx* QTL regions; favorable and unfavorable marker resistance haplotypes are indicated in green and red, respectively, rare and missing haplotypes are presented in grey and white, respectively. Adjusted means of each pea line for ^c^ field resistance synthetic variables and isolate- specific variables in controlled conditions, as well as ^g^ flowering and morphological synthetic variables in healthy nursery; names of variables are described in **Supplementary Table 4**. Number of favorable and unfavorable haplotypes for: ^d^ resistance to *A. euteiches* and ^f^ flowering or morphological traits, for each pea line.
