# Supplementary material for: Advanced backcross QTL analysis and comparative mapping with RIL QTL studies and GWAS provide an overview of QTL and marker haplotype diversity for resistance to Aphanomyces root rot in pea (Pisum sativum)
Source: Front Plant Sci. 2023 Sep 28;14:1189289. doi: 10.3389/fpls.2023.1189289 (PMC10569610; doi:10.3389/fpls.2023.1189289)
Supplement: Supplementary Figure 1 — Frequency distribution of EMMs obtained for A. euteiches resistance and flowering variables in the (A) Eden x E11 and (B) Eden x LISA AB populations. Scoring variables are coded as presented in Supplementary Table 1 . Reference susceptible Eden and partially resistant E11 and LISA parents are indicated in red and green, respectively. n: total number of pea lines assessed; m: mean ± standard deviation; H2: mean-based heritability. [file DataSheet_1.pdf]

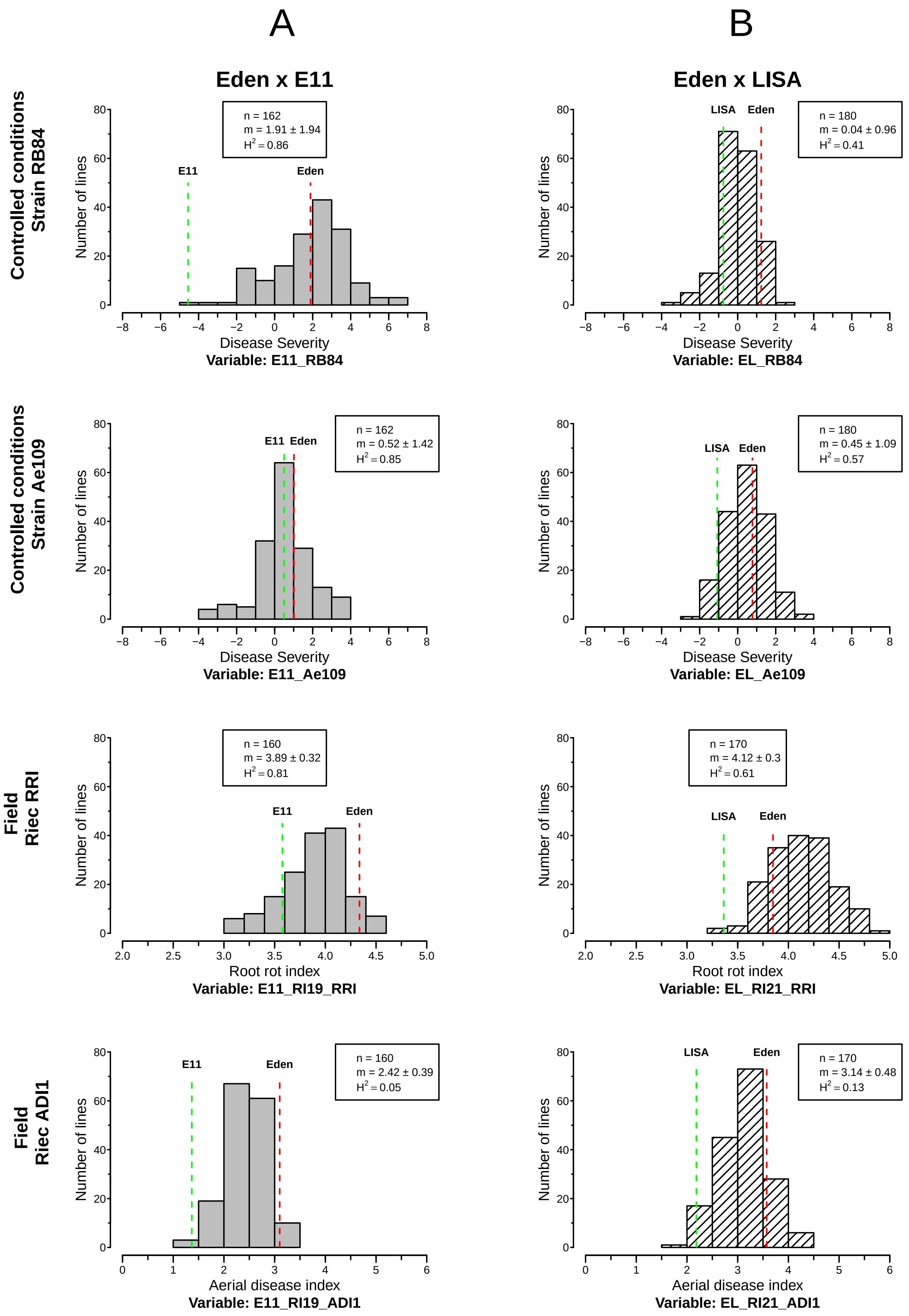
A
B
Eden x E11
Eden x LISA
Controlled conditions
Strain RB84
Controlled conditions
Strain Ae109
Field
Riec RRI
Field
Riec ADI1
Number of lines
n = 162
m = 1.91 ± 1.94
H2 = 0.86
E11
Eden
Disease Severity
Variable: E11_RB84
LISA
Eden
n = 180
m = 0.04 ± 0.96
H2 = 0.41
Disease Severity
Variable: EL_RB84
E11 Eden
n = 162
m = 0.52 ± 1.42
H2 = 0.85
Disease Severity
Variable: E11_Ae109
LISA Eden
n = 180
m = 0.45 ± 1.09
H2 = 0.57
Disease Severity
Variable: EL_Ae109
n = 160
m = 3.89 ± 0.32
H2 = 0.81
E11
Eden
Root rot index
Variable: E11_RI19_RRI
n = 170
m = 4.12 ± 0.3
H2 = 0.61
LISA
Eden
Root rot index
Variable: EL_RI21_RRI
E11
Eden
n = 160
m = 2.42 ± 0.39
H2 = 0.05
Aerial disease index
Variable: E11_RI19_ADI1
LISA
Eden
n = 170
m = 3.14 ± 0.48
H2 = 0.13
Aerial disease index
Variable: EL_RI21_ADI1

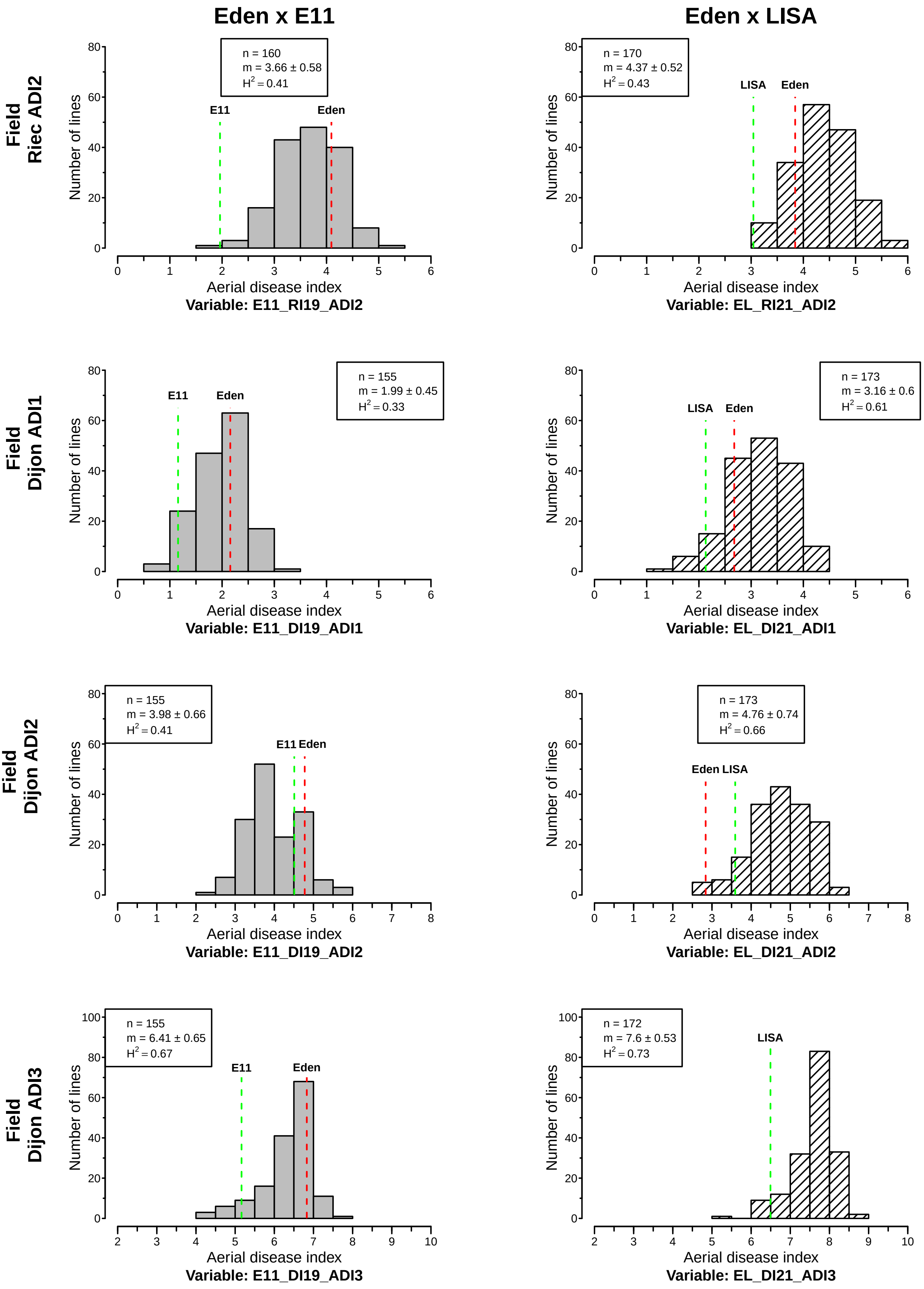
Eden x E11
Eden x LISA
Field
Riec ADI2
Field
Dijon ADI1
Field
Dijon ADI2
Field
Dijon ADI3
n = 160
m = 3.66 ± 0.58
H2 = 0.41
E11
Eden
Number of lines
Aerial disease index
Variable: E11_RI19_ADI2
n = 170
m = 4.37 ± 0.52
H2 = 0.43
LISA
Eden
Variable: EL_RI21_ADI2
n = 155
m = 1.99 ± 0.45
H2 = 0.33
Variable: E11_DI19_ADI1
n = 173
m = 3.16 ± 0.6
H2 = 0.61
Variable: EL_DI21_ADI1
n = 155
m = 3.98 ± 0.66
H2 = 0.41
Variable: E11_DI19_ADI2
n = 173
m = 4.76 ± 0.74
H2 = 0.66
Variable: EL_DI21_ADI2
n = 155
m = 6.41 ± 0.65
H2 = 0.67
Variable: E11_DI19_ADI3
n = 172
m = 7.6 ± 0.53
H2 = 0.73
Variable: EL_DI21_ADI3

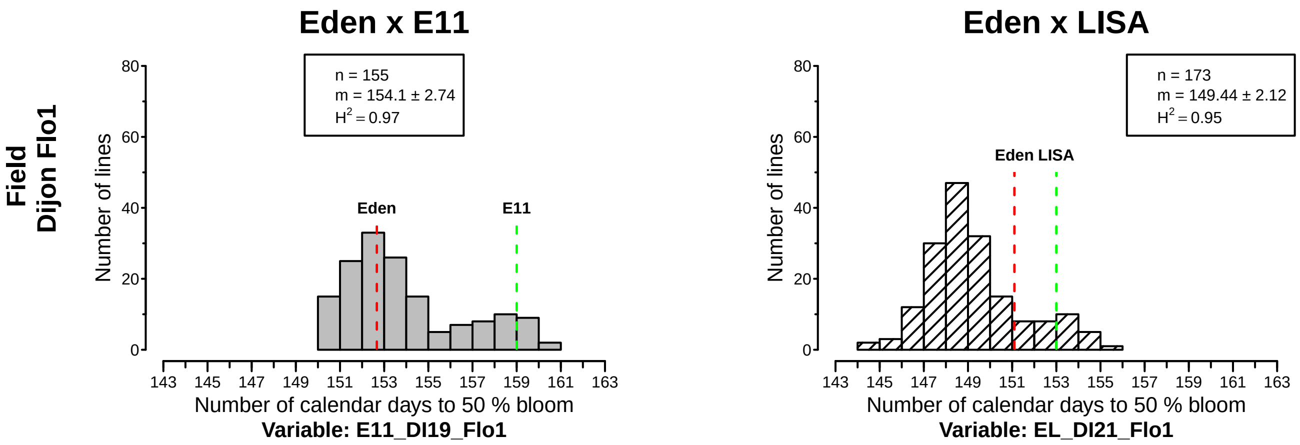
Field
Dijon Flo1
Eden x E11
n = 155
m = 154.1 ± 2.74
H2 = 0.97
Eden
E11
Number of lines
0
20
40
60
80
143
145
147
149
151
153
155
157
159
161
163
Number of calendar days to 50 % bloom
Variable: E11_DI19_Flo1
Eden x LISA
n = 173
m = 149.44 ± 2.12
H2 = 0.95
Eden LISA
Number of lines
0
20
40
60
80
143
145
147
149
151
153
155
157
159
161
163
Number of calendar days to 50 % bloom
Variable: EL_DI21_Flo1
