# Supplementary material for: Advanced backcross QTL analysis and comparative mapping with RIL QTL studies and GWAS provide an overview of QTL and marker haplotype diversity for resistance to Aphanomyces root rot in pea (Pisum sativum)
Source: Front Plant Sci. 2023 Sep 28;14:1189289. doi: 10.3389/fpls.2023.1189289 (PMC10569610; doi:10.3389/fpls.2023.1189289)
Supplement: Supplementary Figure 2 — (A) LD decay in the pea-Aphanomyces collection. Colored curves represent the estimated LD decay for each LG. Dashed vertical lines represent the LD threshold (maximum r2/2) and arrows the LD decay rate, as the estimated genetic distance (cM) to reach this LD threshold on each LG. (B) Population structure in the pea-Aphanomyces collection for 10 subgroups (Q). Each colored horizontal line of individual accession shows the ancestral fraction that was assigned proportionally to the estimated clusters. (C) Ward’s clustered heatmap of the kinship matrix of the pea-Aphanomyces collection. The color gradient represents the degree of relationship between two lines. Pea lines are gathered in 14 subgroups described in the legend. [file DataSheet_2.pdf]

A

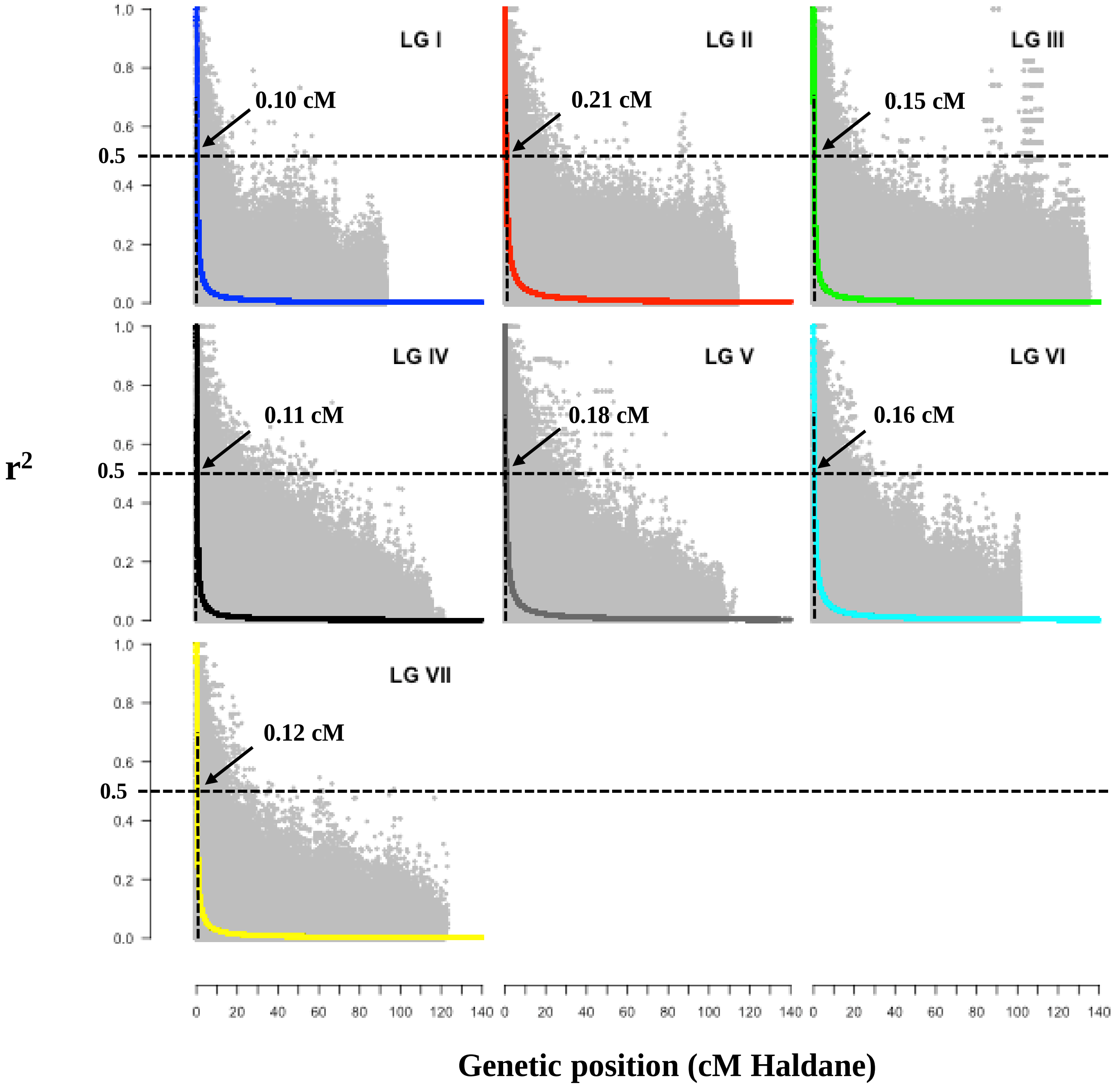
LG I
LG II
LG III
LG IV
LG V
LG VI
LG VII
0.10 cM
0.21 cM
0.15 cM
0.11 cM
0.18 cM
0.16 cM
0.12 cM
0.5
r2
Genetic position (cM Haldane)

B

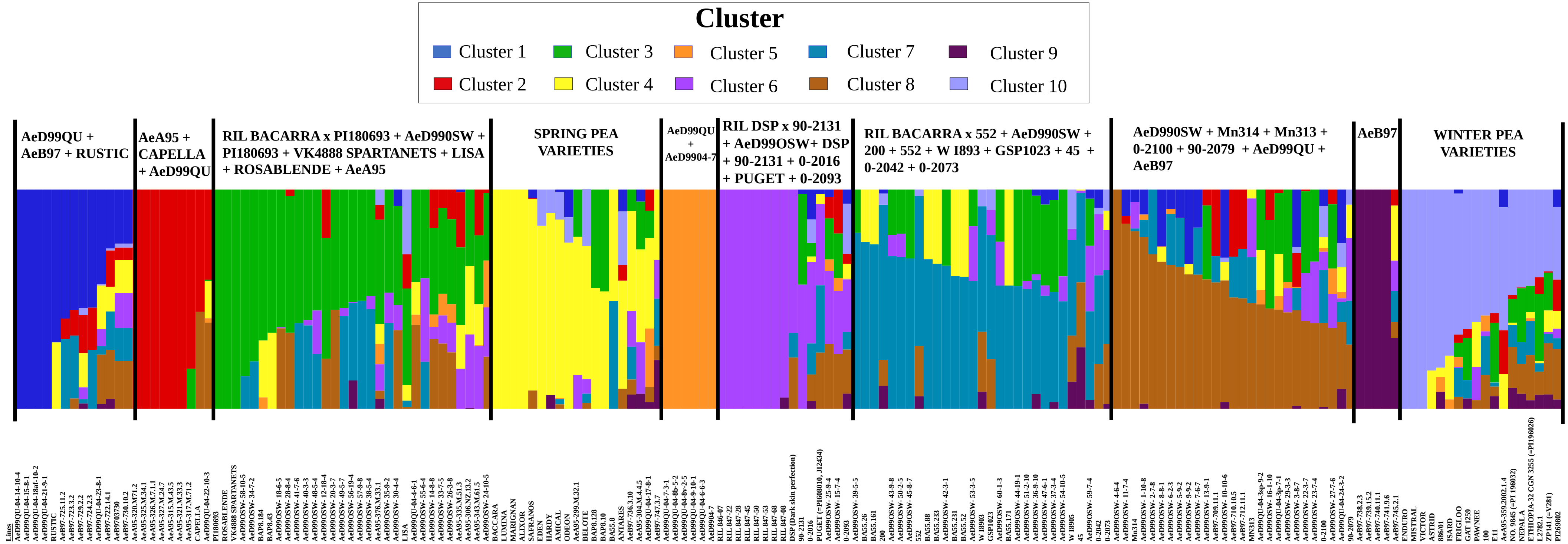
Cluster
Cluster 1
Cluster 2
Cluster 3
Cluster 4
Cluster 5
Cluster 6
Cluster 7
Cluster 8
Cluster 9
Cluster 10
AeD99QU + AeB97 + RUSTIC
AeA95 + CAPELLA + AeD99QU
RIL BACARRA x PI180693 + AeD990SW + PI180693 + VK4888 SPARTANETS + LISA + ROSABLENDE + AeA95
SPRING PEA VARIETIES
AeD99QU + AeD9904-7
RIL DSP x 90-2131 + AeD99OSW+ DSP + 90-2131 + 0-2016 + PUGET + 0-2093
RIL BACARRA x 552 + AeD990SW + 200 + 552 + W I893 + GSP1023 + 45 + 0-2042 + 0-2073
AeD990SW + Mn314 + Mn313 + 0-2100 + 90-2079 + AeD99QU + AeB97
AeB97
WINTER PEA VARIETIES

C

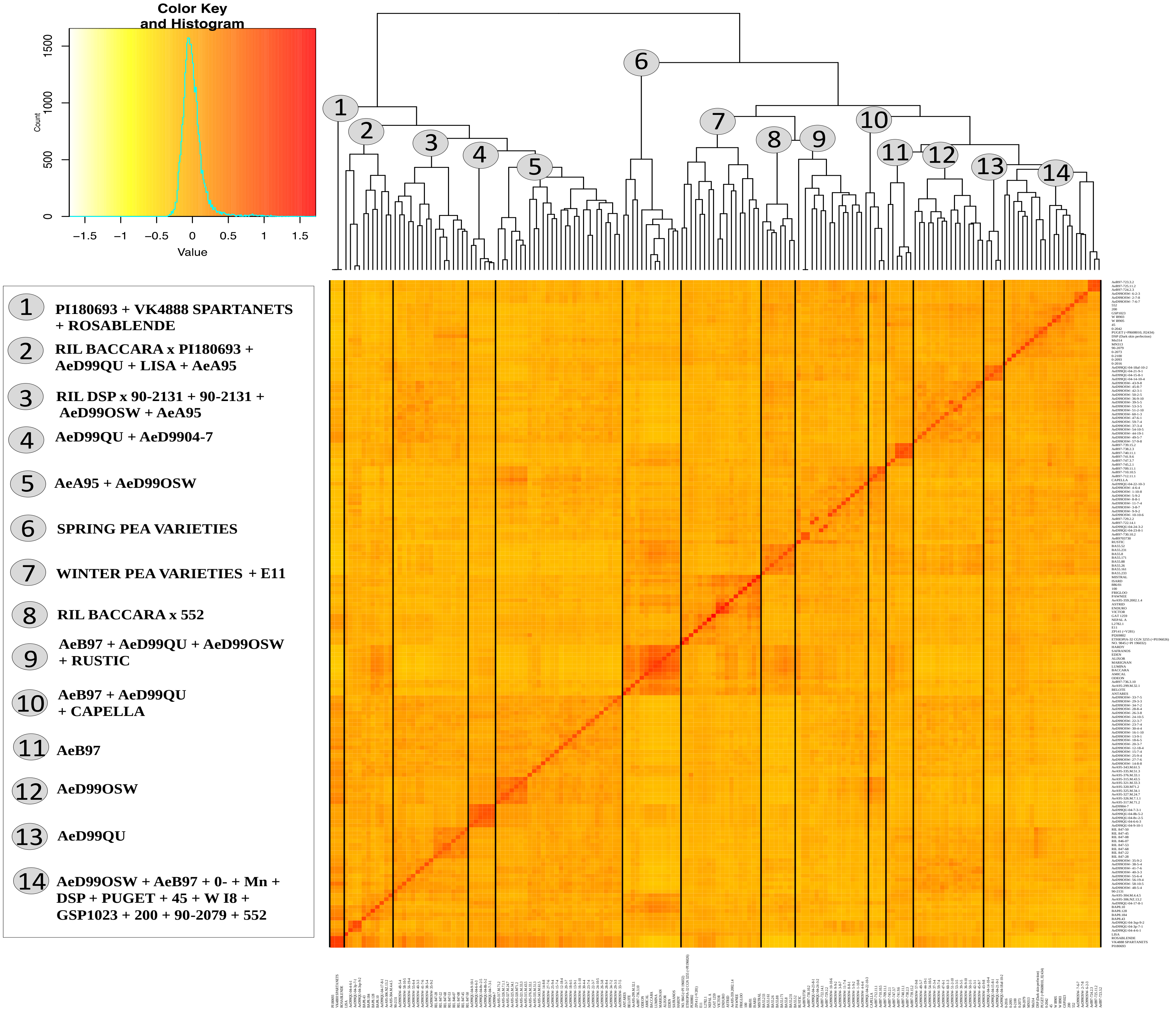

Color Key
and Histogram
Count
Value
1 PI180693 + VK4888 SPARTANETS + ROSABLENDE
2 RIL BACCARA x PI180693 + AeD99QU + LISA + AeA95
3 RIL DSP x 90-2131 + 90-2131 + AeD99OSW + AeA95
4 AeD99QU + AeD9904-7
5 AeA95 + AeD99OSW
6 SPRING PEA VARIETIES
7 WINTER PEA VARIETIES + E11
8 RIL BACCARA x 552
9 AeB97 + AeD99QU + AeD99OSW + RUSTIC
10 AeB97 + AeD99QU + CAPELLA
11 AeB97
12 AeD99OSW
13 AeD99QU
14 AeD99OSW + AeB97 + 0- + Mn + DSP + PUGET + 45 + W I8 + GSP1023 + 200 + 90-2079 + 552
